# A Review on Sulfonated Polymer Composite/Organic-Inorganic Hybrid Membranes to Address Methanol Barrier Issue for Methanol Fuel Cells

**DOI:** 10.3390/nano9050668

**Published:** 2019-04-28

**Authors:** Duraibabu Dhanapal, Min Xiao, Shuanjin Wang, Yuezhong Meng

**Affiliations:** The Key Laboratory of Low-Carbon Chemistry & Energy Conservation of Guangdong Province/State Key Laboratory of Optoelectronic Materials and Technologies, School of Materials Science and Engineering, Sun Yat-Sen University, Guangzhou 510275, China; duraibb@gmail.com (D.D.); stsxm@mail.sysu.edu.cn (M.X.)

**Keywords:** sulfonated polymer composites, organic membrane, inorganic membrane, organic–inorganic hybrid membrane, fuel cell

## Abstract

This paper focuses on a literature analysis and review of sulfonated polymer (s-Poly) composites, sulfonated organic, inorganic, and organic–inorganic hybrid membranes for polymer electrolyte membrane fuel cell (PEM) systems, particularly for methanol fuel cell applications. In this review, we focused mainly on the detailed analysis of the distinct segment of s-Poly composites/organic–inorganic hybrid membranes, the relationship between composite/organic– inorganic materials, structure, and performance. The ion exchange membrane, their size distribution and interfacial adhesion between the s-Poly composites, nanofillers, and functionalized nanofillers are also discussed. The paper emphasizes the enhancement of the s-Poly composites/organic–inorganic hybrid membrane properties such as low electronic conductivity, high proton conductivity, high mechanical properties, thermal stability, and water uptake are evaluated and compared with commercially available Nafion^®^ membrane.

## 1. Introduction

**Figure 1:**
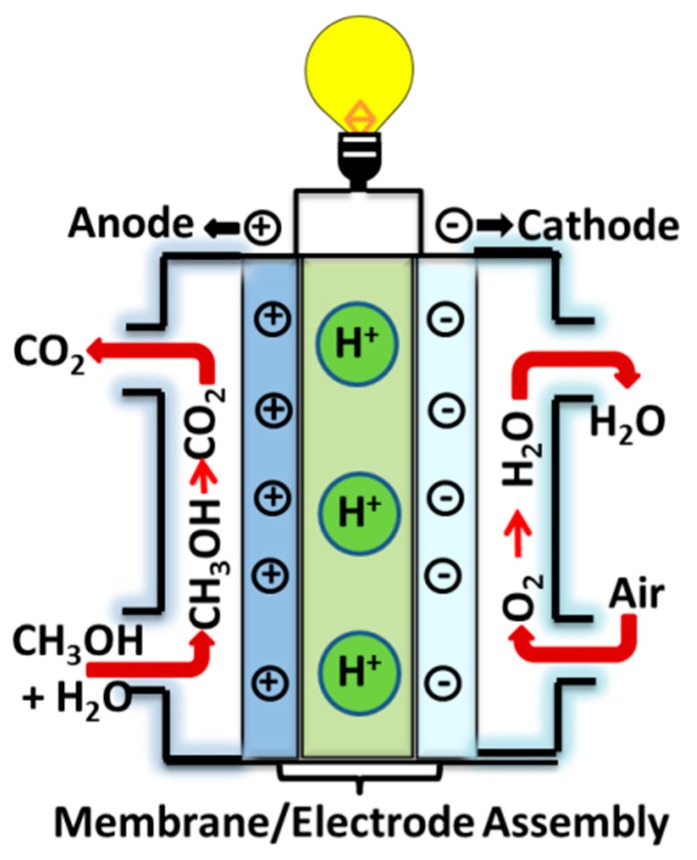
Schematic representation of anodic and cathodic reactions in a typical fuel cell.

The fossil fuel sources are important to the auxiliary environmental pollution, and climate changes exist in the worldwide which need to discover eco-friendly and renewable alternative fuels [1,2]. Fuel cells are direct conversions of chemical energy into electrical energy, and have advantages such as environmental friendliness, high power density, noiseless manipulation, and low emission of pollutants [3,4,5]. The reaction mechanism involved in fuel cells is electrochemical devices, whereas a hydrogen-based chemical acts as an anode, generating protons and electrons in the presence of a platinum catalyst. An electrically insulating electrolyte allows protons to pass through to the cathode and blocks the electrons, assisting the current flow in the external circuit. At the cathode, electrons are re-combined with protons and oxygen provided at the cathode to produce water (Figure 1). The major advantages of fuel cells are that (i) no generation is harmful (SO_x_, NO_x_, CO_x_, and CO); (ii) higher efficiency; and (iii) reduced sound pollution. Nevertheless, drawbacks include storage, processing for fuel cells in the automobile, transportation, and portable applications [6,7]. Therefore, hydrogen-based fuel systems require new technology, a new infrastructure for fuel storage, and delivery and handling safety, respectively. At this moment, liquid fuel can prominently simplify handling and significantly increase the energy density of the fuels [8]. Recently, direct methanol fuel cells (DMFCs) are promising in the field of vehicular transportations owing to the highest energy conversion efficiency, and environmental safety, consumer electronics, and backup of power [9,10,11,12,13]. 

**Figure 2:**
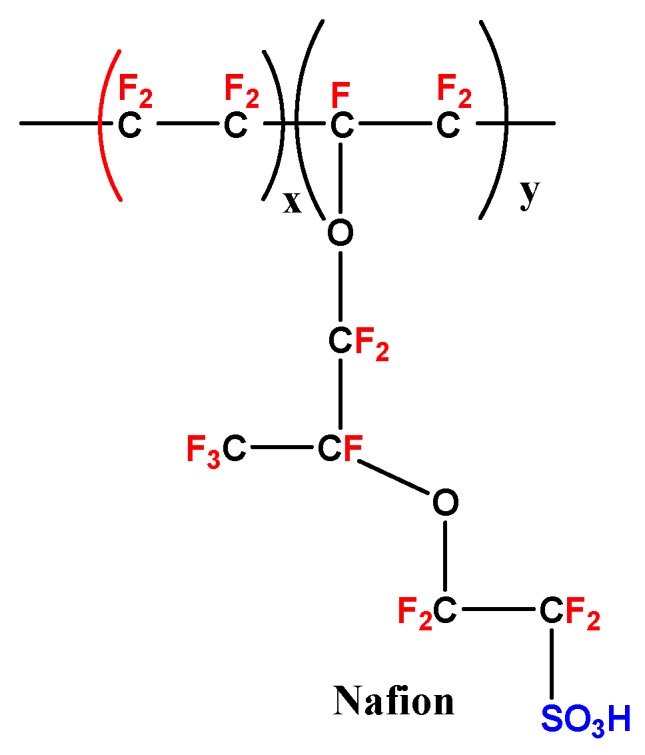
Chemical structure of Nafion^®^.

**Figure 3:**
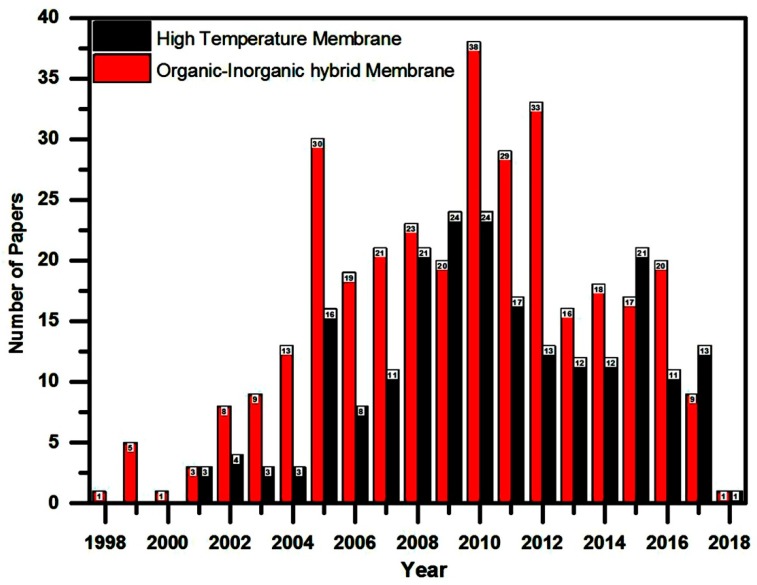
Bar chart illustration of the number of papers published per year 1998–2018 analyzed by Scopus database.

Especially in portable applications, there are several advantages, such as storage easy, high energy density, low cost of methanol, low emission, and direct oxidation of methanol without the reforming process [14,15,16]. At present, this research is mainly focused on the DMFCs; there are two methods which are generally applied in the DMFCs’ performance as follows: the exchange of cations (proton) and the exchange of anion (hydroxyl) [17,18,19,20,21]. However, some difficulties in the DMFCs’ process such as the methanol kinetics oxidation are relatively slow on the anode catalyst, the catalysts produce CO, which affect the electrode process at low temperatures, parasitic crossover methanol and, high cost membranes [22,23,24,25,26]. A new type of improved electro catalyst and electrolyte membranes was prepared which is based on DMFCs applications to overcome the flaws [27,28]. Nowadays, commercially available Nafion^®^ (Figure 2) type perfluorinated polymer versatile polymer electrolyte membrane (PEM) fuel cell materials are widely accepted as DMFCs due to the high levels of electrochemical stability, mechanical strength, proton conductivity, etc. However, the main drawbacks of Nafion^®^ such as high cost, high methanol crossover, difficulty in synthesizing and processing restrict their use in high-performance applications [29,30]. Moreover, the Nafion^®^ membrane possesses an excellent Proton-Exchange Membrane Fuel Cell (PEMFC) optimization at an approximate temperature (~≤80 °C). Unfortunately, the Nafion^®^-based membrane electrode assemblies (MEAs) are poor, when the temperature decreases, as well as increases above 100 °C, it can affect the loss of humidity, reduce proton conductivity, electrochemical stability, and mechanical strength of the PEM at high temperatures. In this connection, to prepare a PEM based on Nafion^®^ membrane was reinforced with varying types of modified composites. The multilayer membranes have been developed to reduce the methanol crossover without deterioration of the proton conductivity owing to higher temperature capability, and thermo oxidative and chemical stability performance [31,32,33,34,35,36,37,38]. On the other hand, the sulfonated polyarylene ether ketones (SPAES), sulfonated polysulfone (SPSU), and sulfonated polyimide (SPI) are widely used as a proton conductive polymer in DMFCs [39,40,41]. Furthermore, the abovementioned sulfonated polymer (s-Poly) materials are relatively cost-effective with high proton conductivity but poor chemical resistance under the DMFCs’ operation conditions. Consequently, the incorporation of inorganic fillers, e.g., silica, titania and zirconia with Nafion^®^ and other polymers such as poly vinylidene fluoride (PVDF), poly 2-acrylamido-2-methyl propanesulfonic acid (AMPS), and polypyrrole, are used to increase resistance in fuel permeation. The results suggest that the additions of higher amounts of inorganic fillers are utilized for the tendency of agglomeration in inorganic and within the polymer, which lead to incompatibility between the fillers and the polymer matrix [42,43,44]. Among them, polymer compositing is the one of the most effective approaches to improving the stability of proton conductivity polymer, with non-conductive engineering polymers having the outstanding mechanical strength and high chemical stability. Generally, the mixing of two or more distinct polymers showed the enhancement of the superior properties in the polymer composite [45]. The long-term record of published literature on sulfonated polymeric composites, organic and inorganic hybrid membranes are presented in Figure 3. In present review work, we mainly focused on the overview of the newly developed s-Poly composite and organic–inorganic hybrid membranes for fuel cell applications. Furthermore, this study highlights both past and present work on various methods of the s-Poly composite and organic–inorganic hybrid membrane materials used. The performances of the composite membranes are summarized and discussed in detail; the physical–chemical and electrochemical properties of modified composite membranes are compared with those of the neat Nafion^®^ membrane. The review indicates significant prospects for the imminent application of direct methanol fuel cell applications and has triggered the efforts on studies of atomic layer deposition. 

## 2. Composite Membranes

### 2.1. Sulfonated Composite Membranes

**Figure 4:**
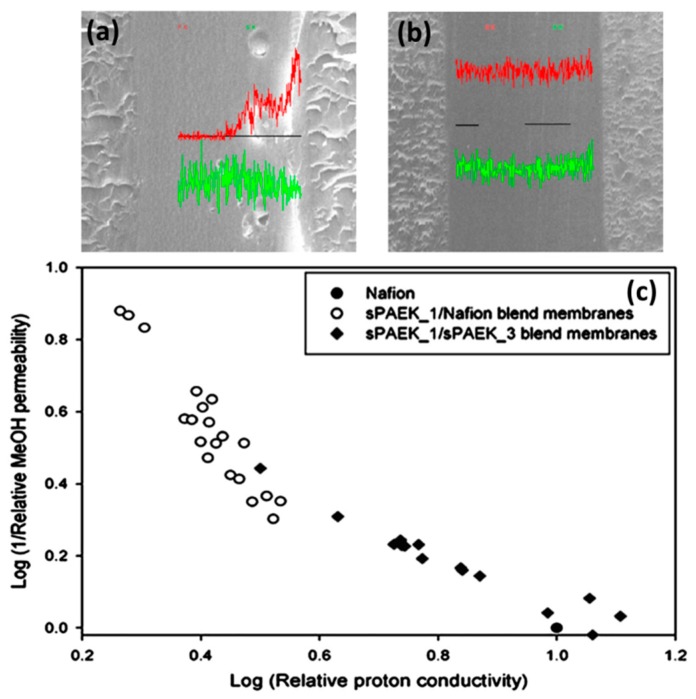
(**a**) Cross-sectional morphology of sPAEK 1:Nafion^®^ = 2:1 by scanning electron microscopy (SEM) and Energy-dispersive X-ray analysis (EDAX); (**b**) Cross-sectional morphology of sPAEK 1:sPAEK 3 = 2:1 by SEM and EDAX; (**c**) Summary of proton conductivity and methanol permeability of blend membranes. Reproduced with permission from [55]. Copyright Elsevier, 2008.

In the present scenario, Nafion^®^ membranes have been widely used in the applications of proton exchange membrane fuel cells (PEMFCs). However, these PEMFCs are commercially available in Nafion^®^ membranes is their low methanol crossover, high cost, and limitations when operating at high temperatures for DMFC applications. To overcome flaws in PEMFCs, sulfonic acid groups in the polymer backbone for the improvement of the membrane’s properties such as high proton conductivity and high temperature performance at low cost [46,47]. Some common used s-Polys are sulfonated poly (benzimidazole), sulfonated polyimidies, sulfonated poly (phenylene ether ether sulfone), sulfonated polystyrene, sulfonated poly (ether ether ketone), and sulfonated poly (arylene ether sulfone) etc. [48,49,50,51,52,53]. Furthermore, the addition of excess amounts of sulfonic acid moiety inside the backbone of the polymer membrane leads to reduced mechanical, swelling properties and higher water uptake, and low dimensional stability under wet/dry conditions, respectively [54]. In a recent study, Choi et al. [55] prepared blend membranes sulfonated poly arylene ether ketone (sPAEK)/Nafion^®^ and sPAEK/sPAEK for DMFCs. The methanol permeability, water uptake, and proton conductivity of the sulfonated blend membranes (sPAEK/Nafion^®^ and sPAEK/sPAEK) properties were analyzed by the phase separated morphology, as depicted in Figure 4. The authors observed that the sulfonated blend membranes (sPAEK/Nafion^®^) has the same values of sPAEK due to a rich layer thickness of sPAEK (Figure 4). Moreover, the sPAEK/sPAEK blend membranes show in-between the properties of sPAEK/Nafion^®^ and sPAEK; this could be due to the variations in the surface morphology. Among them, sPAEK/sPAEK blend membranes exhibit lower methanol permeability, high proton conductivity other than Nafion^®^. Hence, sPAEK/sPAEK blend membranes can be substituted for Nafion^®^. 

**Figure 5:**
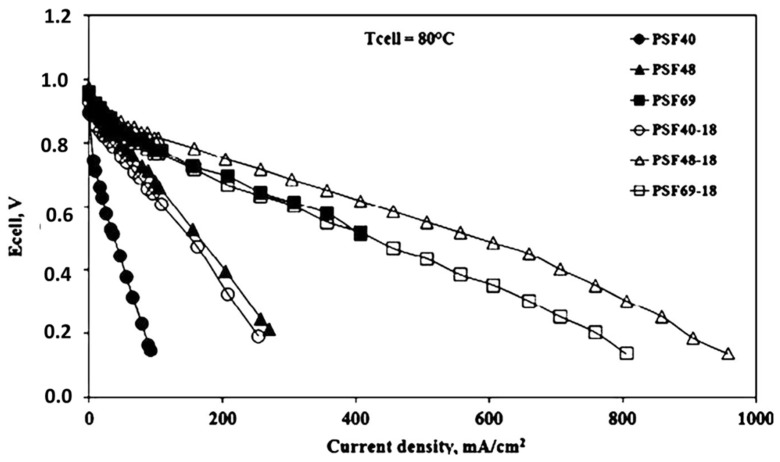
I–V profiles for s-PSF and PSF-18 membranes at 80 °C. Reproduced with permission from [57]. Copyright Elsevier, 2013.

A study by Bi et al. [56] prepared sulfonated non-fluoropolymers: cross-linked with sulfonated polyimide (SPI) and cross-linked sulfonated poly arylene ether sulfone (cSPAES). The stability of the sulfonated blend membranes in water and methanol solutions were a greater improvement over the introduction of SPI. The s-Poly blend membranes showed slightly lower proton conductivity properties when compared with the cSPAES due to the addition of SPI. The proton conductivity value of cSPAES70–5/SPI(5/5) at 50% relative humidity (RH) in water is around 1.7 times better than one half of Nafion^®^ 112 (Table 1). The author concludes that the s-Poly membranes exhibited good high proton conductivity, mechanical, and thermal property which leads to promising results in fuel cell applications. Pedicini et al. [57] analyzed the sulphonated polysulphone (SPSF) containing urethane groups can improve the proton conductivity of a polymer electrolyte fuel cell (PEFC) at a temperature of 80 °C. The SPSF with a sulfonation degree (DS), ranging 40–70% was prepared standardized procedure. An aliphatic polyurethane (PU) diol in oligomeric was added to the SPSFs with different sulfonation levels to prepare composite membranes, containing 18 wt % of PU, casting by using the doctor-blade technique. The amount of 18 wt % with PU and the ion exchange capacity (IEC) values slightly increased with respect to the pristine polymers due to the ionic cross-linking to the slight increase in the water uptake values were observed for each DS between the pristine and corresponding composite. All prepared samples showed a lower swelling than the critical value of 2 at 100 °C. These results suggested that the enhancement of the proton conductivity for each DS, which was due to the interaction between the sulfonic and nitrogenous groups caused by tautomerization mechanism. This study confirmed that the composite membranes’ showed better performance than the bare membranes (Figure 5). In addition, the best performance was observed in the PSF48-18 content of sulfonated composite membranes showed better performance between the electrochemical, physical, and chemical properties. 

**Figure 6:**
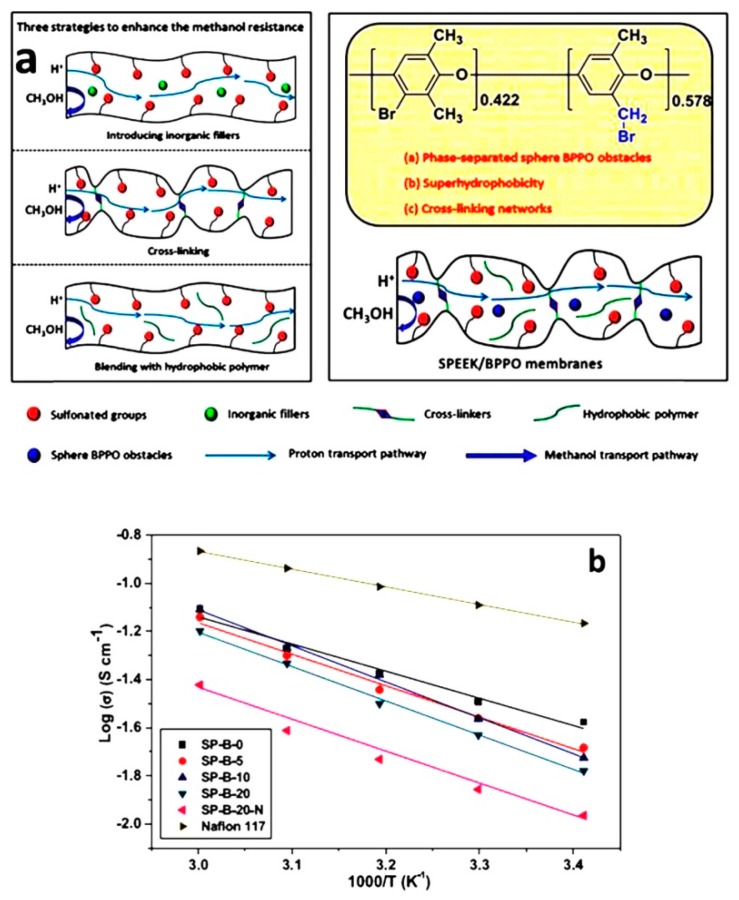
(**a**) Schematic illustration of BPPO’s functions of the super hydrophobic BPPO filler toward the neat methanol operation of DMFC. (**b**) Proton conductivity of SPEEK/BPPO blend membranes. Reproduced with permission from [59]. Copyright Elsevier, 2017.

Zhao et al. [58] prepared sulfonated poly (ether ether ketone) (SPEEK)/polybenzoxazine (PBa) by cross-linked composites for DMFCs applications. The SPEEK membranes were catalyzed by the ring-opening polymerization of benzoxazine (Ba). The formation of PBa cross-linked membrane was established with the help of monomers. The cross-linked (SPEEK/PBa) structure provides better mechanical integrity, thermal stability, and dimensional stability than the SPEEK membrane. The author found that the SPEEK/PBa-15% and SPEEK/PBa-20% blend membranes exhibited stable properties in the DMFCs applications. Moreover, the proton conductivity properties of the sulfonated blend (SPEEK/PBa) membranes decreased with increasing content (15–25%) of PBa. The proton conductivity of the SPEEK/PBa-15% blend membrane initially exhibited 4.25 × 10^−3^ S cm^−1^ at 30 °C, and dramatically increased 2.46 × 10^−2^ S cm^−1^ at 80 °C, these variations may be due to the impact of stimulation of the thermal proton transport process and segmental motion modifications in the polymer (Table 1). On the other hand, the methanol coefficient diffusion of the SPEEK/PBa-15% blend membrane reduced by 93% higher than the Nafion^®^ 117 membranes, and the coefficient values of sulfonated blend membranes is presented in Table 1. Hence, it was suggested that the selectivity maximum at 80 °C is 4.45 × 10^3^ Ss cm^−3^ (SPEEK/PBa-15%), which is 1.3 times higher than Nafion^®^ 117 membranes. From the result, it was observed that the SPEEK/PBa blend membranes showed excellent properties, and this can be due to the cross-linking between SPEEK/PBa and the rigid backbone of PBa. A multifunctional brominated sulfonated blend membrane fabricated Liu et al. [59], a novel PEM of sulfonated SPEEK membrane, is modified by the integration of super hydrophobic bromomethylated poly phenylene oxide (BPPO). The methanol permeability and proton conductivity of SPEEK/BPPO membrane decreased gradually with increased content of BPPO (Figure 6a). Particularly, for the SP-B-20 membrane, the values found to be lowest methanol permeability (1.39 × 10^−7^ cm^2^ s^−1^) and reduced proton conductive properties at 60 °C (0.064 S cm^−1^). This could be attributed to the cross-linked networks (Friedal–Craft reaction) between the nucleophile phenyl rings and bromobenzyl. Further, the hydrophobic nature of BPPO’s polymeric backbone and the formation of BPPO’s spherical obstacles. Consequently, the high relative selectivity values obtained from SPEEK/BPPO membranes compared to the SPEEK membrane show the highest value of 11.98 × 10^4^ Ss cm^−3^ for SP-B-20, which is more than two-times the value of Nafion^®^ 117. This may be due to the SPEEK/BPPO membrane demonstrating a much higher cell performance at the low methanol concentration and high methanol concentration (Figure 6b). The results suggest that the SPEEK/BPPO membranes are suitable for high methanol concentration in DMFCs applications.

### 2.2. Sulfonated Fluoride Composite Membranes

**Figure 7:**
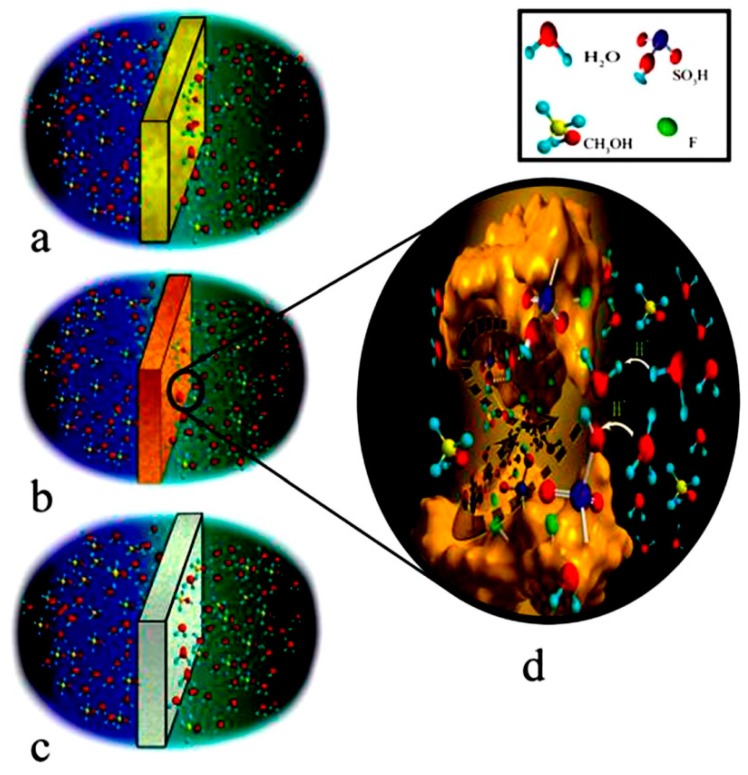
Schematic presentation of water and methanol crossover from (**a**) MS, (**b**) MSSPx, and (**c**) MSPx membranes, and (**d**) proton transferring through the membrane. Reproduced with permission from [73]. Copyright Royal Society of Chemistry, 2016.

**Figure 8:**
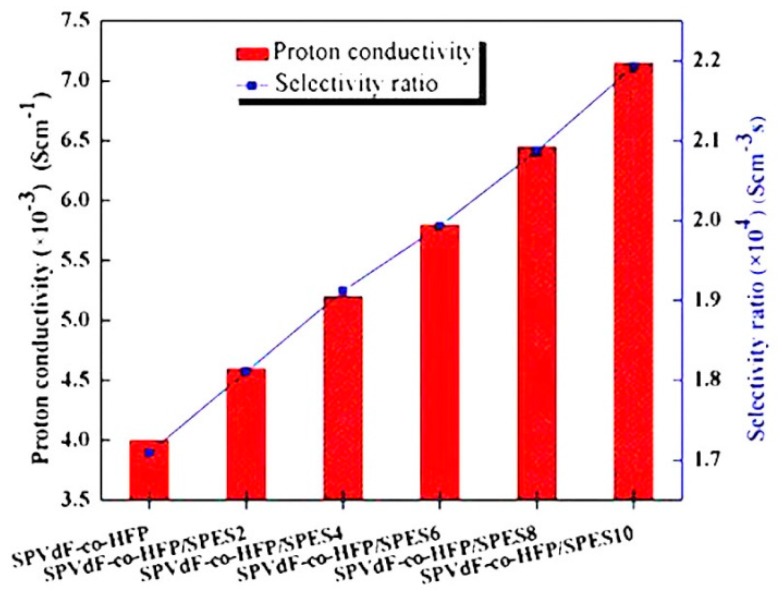
Proton conductivity and selectivity ratio of SPVdF-*co*-HFP and SPVdF-*co*-HFP/SPES blend membranes. Reproduced with permission from [74]. Copyright Elsevier, 2017.

Fluorinated polymers are generally afforded excellent mechanical, thermo-chemical stability due to the C and F chemical bond strength, which contained ion exchange either cationic or anionic membranes to improve the PEM stability through the physical–chemical tuning, cross-linking, thermal treatment, and compositing [60,61,62]. Inan et al. [63] studied the fluorinated polymer of SPEEK/fluorinated blend membrane achievement at low temperature (<80 °C). The sulfonated fluoride blend membranes were prepared by the solution casting method from four different groups of fluorinated polymers: poly vinylidene fluoride (PVDF) with three different molecular weights (180.000, 275.000, 530.000) and PVDF-HFP, Mn: 130.000. The water uptake and proton conductivity value decreased upon addition of fluorinated polymers PVDF with poly vinylidene fluoride-co-hexafluoro propylene (PVDF-HFP) being greater than that of Nafion^®^ 117. The water uptake values of SPEEK70/PVDF (180.000, M_w_: 275.000, M_w_: 530.000) and SPEEK70/PVDF-HFP blend membranes varied between 42.3% and 31%, 43.8–38.3%, 45.7–34.8%, and 45.8–39.9%, respectively. The authors determined that the water uptake value of SPEEK70/PVDF (M_w_ = 275.000) blend membranes was minimized, whereas the methanol permeability value of SPEEK70/PVDF (Mw = 275.000) blend membrane (3.13 × 10^−7^ cm^2^ s^−1^) was much lower than that of Nafion^®^ 117 (1.21 × 10^−6^ cm^2^ s^−1^) (Table 1). The integration of PVDF with SPEEK polymers was responsible for an increase in elongation of the membranes. The increased molecular weight of the PVDF did not show any effect on the Young’s modulus; however, the effect on higher elongation values was observed. Similarly, the increase of the PVDF content in the blend membrane appeared as the lower elongation values at the break. The authors concluded that the best performance was observed for SPEEK70/PVDF (M_w_: 275.000) blend membranes, concerning its high proton conductivity, permeability, chemical stability, and thermal and mechanical properties. Kim et al. [64] studied the casting method in the proton exchange membranes by compositing sulfonated PVDF-HFP and polyethersulfone (PES) in the applications of DMFCs. The authors analyzed the two different polymers such as sulfonated poly arylene ether sulfones (SPAES) as a base polymer and PES/PVDF with varying content (0 to 40 wt %), using N-methyl-2-pyrrolidone (NMP) solvent for the preparation of the SPAES blend membranes. The PVDF/PES blend membranes improvement of the thermal stability as well as decrease in tensile strength properties could be attributed to the poor compatibility between sulfonic acid groups. The methanol permeability of SPAES/PES blend membranes slightly decreased with an increase of PES. However, the SPAES/PVDF blend membranes obtained lower methanol permeability due to the hydrophilic sulfonic acid and the highly fluorinated backbone of PVDF present in the SPAES. The proton conductivity values of the blend membrane showed decreasing patterns with an increasing of PES and PVDF and these changes are attributed to the ionic clusters of the sulfonic acid groups in the cross-linked channels. The authors determined that the SPAES/PES blend membranes exhibited lower methanol permeability and better proton conductivity than the SPAES membranes. The SVDF15 blend membrane showed the highest selectivity (9.09 × 10^6^ Ss cm^−3^) at 25 °C was found to be six times higher due to the decrease of proton conductivity and absence of methanol crossover and values are compared with Nafion^®^. Hereby, we can conclude that these sulfonated composite membranes are suitable for DMFCs applications. Moreover, the study by Unveren et al. [65] prepared the composite membranes based on highly conductive poly1,4-phenylene ether-ether-sulfone (SPEES/PVDF) and characterized in DMFCs applications. The SPEES/PVDF composite membranes decreased water uptake and increased the content of PVDF, which could be the hydrophobic structure of PVDF. The proton conductivity of the SPEES72 membrane was similar at a value of 159.0 mS cm^−1^ at 80 °C and 100% RH compared with Nafion^®^ 115 (133 mS cm^−1^). Nonetheless, the methanol permeability was 50% lower than Nafion^®^ 115 at a temperature of 80 °C. These changes may be due to the addition of 10 wt % PVDF (M_w_: 180,000). The selectivity of results showed better performance than Nafion^®^ 115 and values are given in Table 1. The s-Poly blend membranes exhibited better mechanical and thermal properties, low methanol permeability, and moderate proton conductivity. In another study, Dutta et al. [66] used the partially sulfonated membranes from poly vinylidene fluoride-*co*-hexafluoro propylene (SPVDF-*co*-HFP)/sulfonated polyaniline (SPAni). The sandwiched tri-layered PAni/PVDF-*co*-HFP/PAni, PAni/SPVDF-*co*-HFP/PAni, and SPAni/SPVDF-*co*-HFP/SPAni membranes showed enrichment of transport properties of coated membranes and these properties was compared to SPVDF-*co*-HFP, PVDF-*co*-HFP, Nafion^®^, SPVDF-*co*-HFP/PAni blend, SPVDF-*co*-HFP/SPAni blend, and PVDF-*co*-HFP/PAni blend membranes. The coated tri-layered sandwich membranes showed higher ion exchange capacities, proton conductivities, lower methanol permeability, selectivity, and open-circuit potentials. The 2M methanol aqueous solution at 20 °C was with the coated membranes and it showed the lower methanol permeability values as low as 9.81 × 10^−11^ cm^2^ s^−1^ (PAni/PVDF-*co*-HFP/PAni), 1.72 × 10^−8^ cm^2^ s^−1^ (PAni/SPVDF-*co*-HFP/PAni), and 5.29 × 10^−8^ cm^2^ s^−1^ (SPAni/SPVDF-*co*-HFP/SPAni), respectively. The methanol permeability values are an order of two to four magnitudes lower than the Nafion^®^, pristine, and blend membranes (Table 1) in their turns. The authors observed that the high feed of methanol concentration of the coated tri-layered blend membrane SPAni/SPVDF-*co*-HFP/SPAni’s (105.2 mA cm^−2^ at +0.2 V cell potential and power density of 21.04 mW cm^−2^) exhibited better performance in DMFCs than Nafion^®^ (82.2 mA cm^−2^ at +0.2 V cell potential and a current density of 16.44 mW cm^−2^). Seden et al. [67] synthesized a novel fluorinated blend membrane using Bis (p-hydroxy- tetrafluoro) phenyl) phenyl phosphine oxide (PFPPO-OH) with dichlorodiphenyl sulfone (DCDPS) and Bisfenol-A (copolymer 1a) or 2, 2-bis (4-hydroxyphenyl) hexafluoropropane (Bisphenol-AF) (copolymer 1b). The fluorinated copolymers were blended with SPEEK using a solvent casting method. The addition of varying weight percentages (5, 10, and 20 wt %) in the copolymer showed a decreased proton conductivity with the increase of proton conductivity and water uptake properties. For example, upon addition of 20 wt % of copolymer membranes 1a and 1b, the membrane exhibited the decreased value of proton conductivity and methanol permeability (8.2 × 10^−8^ cm^2^ s^−1^ and 1.3 × 10^−9^ cm^2^ s^−1^) respectively, and these values were compared with Nafion^®^ 117, showing lower values of proton conductivity and methanol permeability (Table 1). Furthermore, the fluorinated polymer properties showed enhanced mechanical and hydrolytic stability, and the chemical structure of phenyl phosphine oxide can increase methanol permeability and thermal stability. Considering all the performance results of the composite membranes with the two novel fluorinated polymers, the authors reported the SPEEK70/Copolymers 1a and 1b sulfonated composite membranes are promising for different types of fuel cell applications. Prasad et al. [68] developed the blended membrane SPEEK and polyvinylidene fluoride-*co*-hexafluoropropylene (PVDF-HFP) filled with Cloisite 30B (C30B). The DMFCs performance was observed at an open circuit voltage (OCV) 0.79 V and a power density maximum of 55 mW cm^−2^. The optimized membrane observed that methanol permeability and selectivity was obtained at 1.35 × 10^−7^cm^2^s^−1^ and 9.63 × 10^4^ Ss cm^−3^ and methanol permeability values were lower than that of Nafion^®^ 117 (Table 1). Besides, the introduction of PVDF-HFP hydrophobicity reduces the density of hydrophilic sulfonic acid groups. The author concluded that the introduction of PVDF-HFP and C30B together can improve the oxidative and dimensional stabilities of PEM. Furthermore, the liquid uptake and the swelling ratios of the membranes decreased with the increasing content of PVDF-HFP. The enhancement of oxidative stability, OCV values, higher selectivity ratios, and thermal and liquid uptake stabilities of the membranes can be suitable for PEMs in DMFC applications. Kumar et al. [69] examined a sulfonated polystyrene blend of PVDF-*co*-HFP/Nafion^®^ which has shown to be capable of PEM characteristics in terms of enriched proton conductivity, water uptake/swelling, maximum power densities, and output current. The methanol permeability and proton conductivity of the S-20 membrane exhibited higher values of methanol and permeability than S-15 and S-30 when compared to Nafion^®^ 117 membranes. The values of proton conductivity and methanol permeability are given in Table 1. In addition, the semi-interpenetrating network (semi-IPN) membrane showed better methanol permeability, oxidative stability, and comparable ion-exchange capacity. A study by Liu et al. [70] analyzed the poly vinylidene fluoride (PVDF) grafted poly styrene sulfonic acid (PVDF-*g*-PSSA) with sulfonated SPEEK. The PVDF-*g*-PSSA copolymer was dispersed homogeneously in the SPEEK matrix with a hierarchical unique particle surface. The blend membranes (SPEEK/PVDF-*g*-PSSA) showed better proton conductivity, lower methanol permeability, and dimensional stability higher than commercially available Nafion^®^ 117. Especially, with a PVDF-*g*-PSSA content of 5 wt %, the composite membrane exhibited the best overall membrane performance, among all the composite membranes (10, 15, and 20 wt % of PVDF-*g*-PSSA), respectively. This could be attributed to the better compatibility and strong interactions between the PVDF-*g*-PSSA and SPEEK matrix. The thermal stability depends upon the water uptake, dimensional change and proton conductivity of the composite membranes. In this work, the authors express the overall performance of SPEEK/PVDF-*g*-PSSA membranes that are produced at low cost and with superior properties, offering great potential as PEMs for DMFCs applications. Das et al. [71] arranged a partially sulfonated SPVDF prepared by incorporating sulfonic acid groups within PVDF, using chlorosulfonic acid as a sulfonating agent. The author prepared a 70/30 wt % (SPVDF/Nafion^®^) composite membrane. The composite membranes are namely SPN-0 (non-sulfonated PVDF/Nafion^®^), SPN-2 (2 h sulfonated PVDF/Nafion^®^), SPN-4 (4 h sulfonated PVDF/Nafion^®^), and SPN-6 (6 h sulfonated PVDF/Nafion^®^). The proton conductivity increased from SPN-0 to SPN-4 with the value of SPN-4 at 3.6 × 10^−2^ S cm^−1^, which is higher than the Nafion^®^ 117. In the case of methanol permeability, SPN-4 composite membranes showed maximum methanol permeability among the all the composite membranes. Likewise, SPN-4 composite membranes exhibited higher membrane selectivity compared to Nafion^®^ 117. The authors claimed that the composite composition exhibited better glass transition temperature, higher storage modulus, higher stress relaxation and superior DMFCs performance at high methanol feed concentration. Mondal et al. [72] studied the modification of Nafion^®^ 117 membranes using the processes of dip-coating. The blend membrane prepared by using polybenzimidazole (PBI) and partially sulfonated polyvinylidinefluoride-*co*-hexafluoro propylene (SPVDF-*co*-HFP). The proton conductivity and methanol permeability of M2 (70/30 SPVDF-*co*-HFP/PBI) coated membranes were higher than M3 (50/50 SPVDF-*co*-HFP/PBI), M4 (30/70 SPVDF-*co*-HFP/PBI), and M5 (PBI)-coated systems, respectively. The results indicated further that M2 membrane showed lower proton conductivity compared to Nafion^®^117 (Table 1). It was observed that the Nafion^®^ 117 membranes coated with 70:30 PBI/SPVDF-*co*-HFP showed better electrical performance (39 mW cm^−2^ at 0.2 V) at a temperature of 90 °C. It was further noted that the coated Nafion^®^ 117 membranes were electrically efficient at high temperatures in DMFCs applications. Bagheri et al. [73] studied a blended membrane, SPEEK/SPVDF-*co*-HFP, prepared by a solution casting method. The SPVDF-*co*-HFP exhibited the finest properties in terms of liquid uptake, swelling, thermal and mechanical stability, methanol permeability, and proton conductivity in DMFC performance. The authors found that the liquid uptake, swelling, methanol permeability, proton conductivity, and thermal and mechanical stability where the best properties, in which 20 wt % (MSSP20) blend (SPEEK/SPVDF-*co*-HFP) membrane exhibited outstanding performances among other blend membranes (MSP10, MSP15, MSP20, MSP25, MSSP15, and MSSP25). Detailed values are given in Table 1 and plots are presented in Figure 7. This may be due to the interaction between PVDF-*co*-HFP and SPEEK blend membranes. Nevertheless, the improved properties of methanol barrier, selectivity, swelling properties, mechanical and thermal stability of SPEEK were observed by adding SPVDF-*co*-HFP and MSSP20 (SPEEK/SPVDF-co-HFP) to produce blend membranes. Devi et al. [74] fabricated SPVDF-co-HFP/SPES composite PEMs for DMFC applications. The PEM composite membranes were prepared by the solution casting method. It was found that the prepared PEMs composite membranes exhibited decreased tensile strength with the addition of hydrophilic sulfonated poly ether sulfone (SPES). Hence, with hydrophilic SPES content increasing, the water uptake, selectivity, and proton conductivity of SPVDF-*co*-HFP/SPES (2, 4, 6, 8 and 10 wt %) composite membranes increased when compared to the neat SPVDF-*co*-HFP (Figure 8). This may due to the hydrophilic regions being round clusters within the polymer matrix which leads to adsorption of water and enhances the proton transport. The authors claimed that the selectivity of the SPVDF-*co*-HFP/SPES (2, 4, 6, 8, and 10 wt %) composite membranes ranged from 1.709 × 10^4^ to 2.193 × 10^4^ Ss cm^−3^, which was much higher than the Nafion^®^ 117 (0.214 × 10^4^ Ss cm^−3^) membrane. Thus, in this case, the SPVDF-*co*-HFP/SPES (2, 4, 6, 8, and 10 wt %) composite membrane water uptake and proton conductivity values were much lower than Nafion 117^®^ (Table 1). 

The sulfonated fluorinated block copolymer (SFBC) was developed by Kim et al. [75] in nucleophilic substitution polymerization and fabricated using the different weight percentages (10 wt %, 20 wt %, and 30 wt %) with phosphotungstic acid (PWA) through the facile solution casting method. The incorporation of PWA into the SFBC-50 the enhancement of mechanical strength, water uptake, proton conductivity, and oxidative stability compared to the neat SFBC-50. For instance, the proton conductivity increased with the increase in temperature by adding PWA to all systems, namely, SFBC-50/PWA-10 (57.93 mS cm^−1^), SFBC-50/PWA-20 (74.15 mS cm^−1^), and SFBC-50/PWA-30 (105.22 mS cm^−1^). This enhancement of mechanical strength, water uptake, proton conductivity and oxidative properties are due to three factors: (i) an excess amount of acidic and –OH groups which acted as high surface PWA; (ii) inherent nature of molecular frameworks of PWA which hold water molecules both physically and chemically; and (iii) the establishment of highly compact pores between the SFBC-50 and PWA. A similar result was observed in the absorbed water uptake values of 14.65% (30 °C) and 60.31% (90 °C) due to high-density hydrophilic functionalities in SFBC-50/PWA-10, SFBC-50/PWA-20 except for SFBC-50/PWA-30. The authors concluded that the incorporation of PWA into the polymer matrix had optimal improvement on the composites’ properties. 

### 2.3. Hydroxyl Sulfonated Composite Membranes

**Figure 9:**
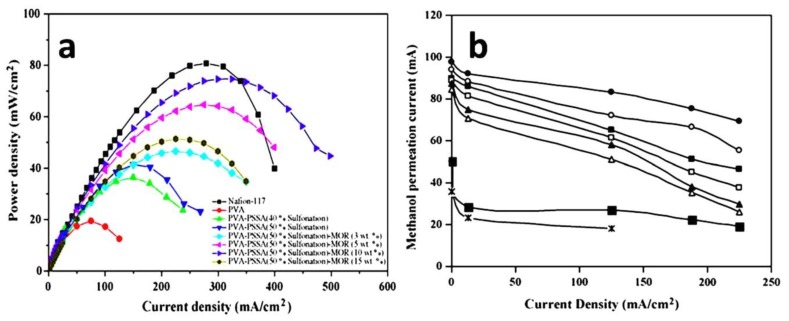
(**a**) Power-density vs. current density for different types of membranes (**b**) Methanol permeability (mA) vs. current density (mA/cm^2^). Symbols: (×) PVA; (○) PVA–PSSA (40% sulfonation); (■) PVA–PSSA (50% sulfonation); (□) PVA–PSSA–mordenite (3 wt %) membrane; (▲) PVA–PSSA–mordenite (5 wt %) membrane; (∆) PVA–PSSA–mordenite (10 wt %) membrane; (■) PVA–PSSA–mordenite (15 wt %) membrane; (●) Nafion-117^®^ membrane. Reproduced with permission from [82]. Copyright Elsevier, 2009.

To enhance membrane performance, the cross-linking of polymers and compositing of poly vinyl alcohol (PVA) has been used to achieve excellency in forming super hydrophilic membranes, and oxidative and hydrolytic stability, to overcome flaws in membrane properties [76,77,78]. Molla et al. [79] studied the effect of SPEEK materials and reported that they are good proton conductors at high temperature (glass transition temperatures of 200 °C) for DMFC application. The addition of PVA membranes at 25 wt % was found to be acceptable for proton conductivity, methanol permeability, and mechanically stability in boiling water. The blend membranes of hydrophobic SPEEK polymers with polyvinyl butyral (PVB) are stable in water at 30 wt %. Additionally, PVA is a hydrophilic polymer which forms a uniform dispersion with SPEEK. In the case of PVB hydrophobic polymer with SPEEK, they showed very low methanol permeability in the phase separation morphology. Hence, the blend membranes SPEEK-35%PVA (IEC = 1.75 meq g^−1^) and SPEEK-30%PVB (IEC = 2.05 meq g^−1^) exhibited reasonably good properties in terms of proton conductivity, mechanical stability, and methanol permeability. The authors further observed that these membranes possess tremendous properties of mechanical and methanol barriers, even though SPEEK shows very low proton conductivities. Furthermore, a study by Lin and Wang et al. [80] observed the PVA/Nafion^®^ (N/VA-b) blend membrane prepared using the casting method in the applications of DMFCs. The methanol permeability, conductivity, and performances of DMFC cell membranes were investigated and compared with Nafion^®^ 117 and Nafion^®^ 212. The results indicated that the blend membranes with a thickness of 50 μm have an optimum PVA content of 10 wt % and 5 wt % for both N/VA-f and N/VA-b membranes, respectively. The single cell tests were demonstrated further in the DMFCs’ performance, which showed the decreased sequence given as N/VA-f > N/VA-b > Nafion^®^ 117 > Nafion^®^ 212. This leads to the N/VA-f having a higher proton conductivity optimum PVA content and better DMFC performance than the N/VA-b membrane. Another study by Yang et al. [81] observed the PVA/SPEEK blend membrane for maintaining the swelling ratio and low methanol permeability in DMFCs was noted further. The composite membranes were prepared using various PVA content by a solvent casting method. The authors attributed the results of thermal stability improvement upon the addition of PVA. This method increased the weight loss temperature of the membranes from 250 °C to 350 °C. The water uptake of the composite membranes increases with increasing the content of PVA, while the absorption of composite membranes in methanol solution shows a decrease upon the addition of PVA. The methanol resistance shows an increase with increasing content of PVA. For instance, S72 (S72 PEEK sulfonated 72 h) shows the highest methanol permeability (1.178 × 10^−5^ cm^2^ s^−1^) when compared to S40 (PEEK sulfonated 40 h), and the lowest value of 5.592 × 10^−6^ cm^2^ s^−1^ in pure SPEEK membranes. In this case of SP72 (PVA:50 wt %), it decreased to 3.588 × 10^−6^ cm^2^ s^−1^, much lower than S40. Owing to the addition of PVA, the membrane brings an outstanding methanol resistance. In addition, the authors indicated that the slightly ion exchange capacity (IEC) and proton conductivity of the composite membranes was due to the specific interaction between SPEEK and PVA molecules. Therefore, from the above mentioned comprehensive explanations, we can conclude that the lower methanol permeability of the composite membranes suggests its potential applications in DMFC. Bhat et al. [82] examined the PVA/polystyrene sulfonic acid (PSSA) composite membranes with mordenite (MOR) and the integration of PVA/PSSA with various degrees of sulfonation. The value of peak power-density of 74 mW cm^−2^ was observed in DMFCs at 50% degree of sulfonation/10 wt % mordenite phase in the PVA/PSSA composite membrane electrolytes. In addition, the methanol crossover current at low 7.5 mA cm^−2^ with 2 M methanol feeds on the DMFCs anode was observed further and shown in Figure 9. Therefore, the author concluded that optimal levels of the composite membrane as the electrolyte and 60% and 46% lower than Nafion^®^ 117/PVA–PSSA membranes in DMFCs. Madaeni et al. [83] studied the non-perfluorinated composite blend membranes, which are prepared by DMFCs based on PVA and poly ether sulfone (PES) via solution casting. The properties of water uptake and swelling degree of the membranes decrease with the increase in phosphotungstic acid (PWA) and the decrease in the amount of PES. During these processes, the IEC was improved by an increased percentage of PVA content. However, the composite membranes of the two polymers did not show any effect on the IEC. The reduced compactness of the membranes and the increase in the free volume was observed by the introducing of PES to PVA. Moreover, the authors claimed that proton conductivity was improved by the addition of PES and an increase in the water content of the membranes was also observed. The performance of membrane electrodes using the highest selective membrane at 80 °C with 1 and 5 M methanol concentrations and oxygen at a pressure of 2 atm in the DMFC was investigated and compared with Nafion^®^ 117 membranes. Furthermore, the homogeneous structure of the composite membranes showed better melting temperature in DSC and the enhancement of thermal stability due to the addition of PES/PVA to the PVA matrix. Yang et al. [84] developed poly vinyl alcohol/sodium alginate membranes (PVASA) by a chemically cross-linking method; PVASA membranes were further treated with glutaraldehyde (GA) as a cross-linking agent to obtain PVASA-GA. The polymer electrolyte membranes were formed by immersing various PVASA membranes in a KOH solution. The PVASA membrane containing KOH showed a decrease thermal property in crystallinity and an increase in melting point with increasing the content of sodium alginate. The ionic conductivity of the PVASA64 membrane at 25 °C (0.091 S cm^−1^, was higher than the modified PVA membranes. They concluded that the effect of cross-linking time on methanol permeability was very significant. It was seen that the selective values of the PVASA82-GA60 membrane at 21.50 × 10^3^ Ss cm^−3^ and a maximum power density of 20.7 mW cm^−2^ were achieved at Ep,max = 0.232 V with a peak current density (*i*_p,max_) of 89.20 mA cm^−2^ at 30 °C for the direct methanol fuel cell. 

Molla and Compan [85] studied SPEEK/PVA composite membranes by infiltration of SPEEK with PVA. This was prepared with water as a solvent and electrospun nanofibers of SPEEK composites with polyvinyl butyral (PVB). They characterized the composite membranes in the applications of DMFCs operating at moderate temperatures greater than 80 °C, and the cross-linking temperature for the SPEEK-PVA system was observed. The hydrated SPEEK-30%PVB nanofibers (6.11 × 10^−4^ S cm^−1^) indicated that the higher proton conductivity in comparison with a dense membrane. Further, the incorporation of the nanofiber mats to the SPEEK-35%PVA matrix provided the enhanced properties of the methanol barrier and proton conductivity with a cross-linking temperature at 120 °C (Table 1). Beydaghi et al. [86] synthesized sulfonated graphene oxide (SGO)/Fe_3_O_4_ nanosheets by a hydrothermal method. The authors incorporated into the composite SPEEK/PVA matrix with a different weight percent of SGO/Fe_3_O_4_ nanosheets. The compositing of PVA/SPEEK membrane shows the increasing trend in tensile strength, water uptake and a decrease trend in proton conductivity and power density. The properties of mechanical stability, proton conductivity and methanol barrier, SGO/Fe_3_O_4_ nanosheets were enhanced by the assimilation of the SPEEK/PVA matrix. The SPEEK/PVA/SGO/Fe_3_O_4_ nanocomposites membrane with optimal nanosheets content (5 wt %) exhibited the low methanol permeability (8.83 × 10^−7^ cm^2^ s^−1^), high tensile strength (51.2 MPa), high proton conductivity (0.084 S cm^−1^ at 25 °C), and high power density (122.7 mW cm^−2^ at an temperature of 80 °C.

### 2.4. Sulfonated Chitosan Containing Composite Membranes

**Figure 10:**
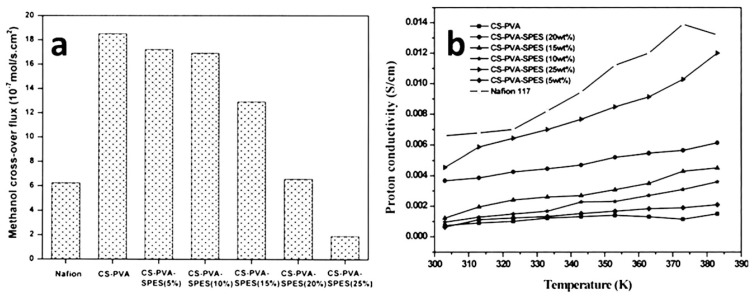
(**a**) Methanol crossover flux for Nafion^®^ 117, CSPVA blend, CS-PVA-SPES (5–25 wt %) mixed-matrix membranes and (**b**) proton conductivity vs. temperature for Nafion^®^117, CS-PVA blend, and CS-PVA-SPES (5–25 wt %) mixed matrix membranes. Reproduced with permission from [89]. Copyright Wiley, 2012.

Biopolymer membranes have a significant effect on polymer electrolytes in the fuel cell environment. These membranes play an important role in the properties of polysaccharide bio resource, biodegradable, chitosan, and biocompatible [87]. Composite s-Poly was developed using the improvement of conductivity of the biopolymer membrane. Muthumeenal et al. [88] introduced a chitosan modified phthaloylation using an excess amount of phthalic anhydride at 130 °C and sulfonated polyethersulfone (SPES) to produce composite membranes. The authors found that the introduction of the phthaloyl group into the chitosan matrix can increase the solubility in organic solvent, film formability, flexibility, methanol permeability, and can be suitable in ion conductivity. The SPES/N-phthaloyl chitosan (NPHCs) membranes exhibit higher conductivity values among the SPES/NPHCs (0.5) and SPES/NPHCs and the methanol permeability of these fabricated membranes is lower than Nafion^®^ (Table 1). The hydrophilic domains are clusters of ionic chains leads to the large absorption of water. This can be accountable for the proton conduction by the hopping mechanism. Moreover, the composite membranes showed better thermal stabilities than Nafion^®^ 117 and relatively high selectivity parameter values indicate the more advantages in DMFC environments. Meenakshi et al. [89] examined the chitosan (CS) and polyvinyl alcohol (PVA) in cross-linked with sulfosuccinic acid (SSA) and sulfonated polyethersulfone (SPES) modified mixed-matrix membranes in DMFCs. The authors discussed that the methanol crossover for these membranes was found to be about 33% lower compared to the Nafion^®^ 117 membranes (Figure 10a). The proton conductivity values showed an increase in the mixed matrix membranes with increasing of SPES from 5 wt % to 25 wt % (Figure 10b). Both cases of water/methanol absorption and ion-exchange capacity (IEC) had a significant effect on membrane conductivity. Further, the proton conductivity for mixed matrix membranes and higher proton conductivity when compared with the Nafion^®^ 117 (Table 1.). The mechanical and thermal properties of mixed-matrix membranes exhibited relatively poor mechanical properties when compared to Nafion^®^ 117. Therefore, it is suggested that the durability of mixed-matrix membranes is lower when compared to Nafion^®^ 117.

Yang and Chiu [90] reported a novel polyvinyl alcohol/chitosan (PCS), the composite membrane prepared by a direct composite process and a solution casting method. The authors studied various composite membranes by immersing in KOH_(aq)_ solution to form a polymer electrolyte membrane, and then the alkaline uptake and swelling ratio in the thickness (*SW_L_*) and plane direction (*SW_A_*) in membranes were measured, respectively. The ionic conductivity and methanol permeability of the membranes showed that the value of methanol permeability through the membrane was lower than that of the Nafion^®^ membrane. From the authors’ experimental data, it can be concluded that when compared to Nafion^®^ and other studies on PCS91-glutaraldehyde (GA) membranes with higher selectivity, these membranes suggest their potential applications in DMFCs. Xiang et al. [91] used chitosan monomers with sulfonic groups, then cross-linked chitosan sulfate with an amino group in the pure chitosan monomers. We can see from the authors’ experimental results that the methanol crossover of composite chitosan membranes and swelling area value decreased from 55.1% to 39.3%. Similarly, the methanol diffusion coefficient decreased to 1.0 × 10^−6^ cm^2^ s^−1^ when compared to pure chitosan at 4.7 × 10^−7^ cm^2^ s^−1^. The content of cross-linked membrane chitosan sulfate around at ∼9.1 wt % chitosan sulfate composite membranes (CCSM 110) showed the best performance on conductivity, the proton conductivity of CCSM 110 enhanced 0.03 S cm^−1^ at 80 °C. Accordingly, the methanol permeability (CCSM10) was much lower than Nafion^®^ 112 (1.9 × 10^−6^ cm^2^ s^−1^). In addition, the thermal analyses of cross-linked sulfate membranes were stable at below 100 °C. It concluded that the CCSM 110 with low methanol permeability is a very important part of DMFCs. 

## 3. Sulfonated-Based Modified Membranes

### 3.1. Sulfonated Organic Membranes

**Figure 11:**
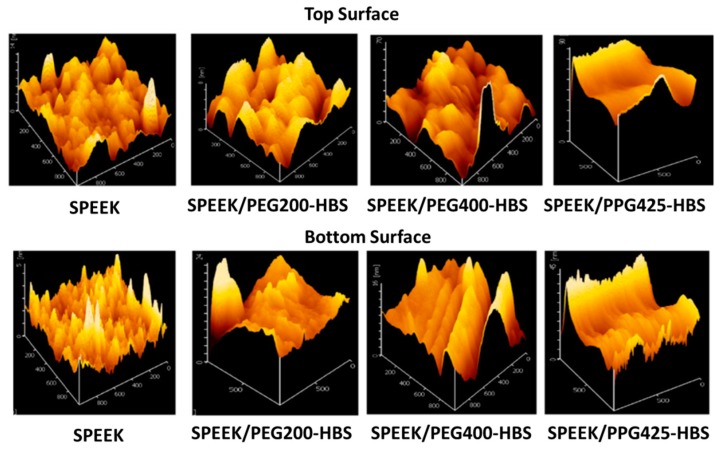
AFM image of nodule structure on membrane surfaces. Reproduced with permission from [96]. Copyright Elsevier, 2009.

**Figure 12:**
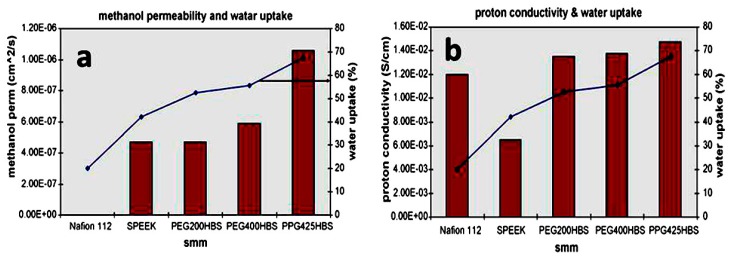
(**a**) Water uptake and methanol permeability of the blended membranes. (**b**) Water uptake and proton conductivity of blended membranes. Reproduced with permission from [96]. Copyright Elsevier, 2009.

Organic compounds generally contain one or more carbon atoms and covalently bond with nitrogen, hydrogen, and oxygen. The polybenximidazole, polyphosphazenes etc., are commonly used polymers in DMFC applications [92,93,94]. These types of polymers composite with other polymer backbone-based carbon or element polymer membranes exhibit excellent chemical stability and good performances. Norddin et al. [95] studied the modification of sulfonated poly ether ether ketone (SPEEK) membranes composites with a charged surface modified macromolecule (cSMM). The SPEEK/cSMM membrane showed lower values of methanol diffusivity (2.75 × 10^−7^ cm^2^ s^−1^) and higher proton conductivity (6.4 × 10^−3^ S cm^−1^) when compared with the SPEEK membrane. The authors mentioned that the transportation of proton domains and increasing water uptake were contributing to the decreased permeability of methanol. Though the authors noted that the thermal and mechanical stability of the composite membrane was slightly reduced when compared to the SPEEK membrane, it was higher than Nafion^®^ 112 membranes. Norddin et al. [96] discussed the effect of modification of the SPEEK membrane by compositing three newly developed charged surfaces modified macromolecules (cSMMs) such as PEG200-HBS, PEG400-HBS, and PPG425-HBS polyols. The authors focused mainly on the surface morphology of the composite membranes and the analyzed the properties of nodule size and rough surface. These changes are attributed to the addition of SO_3_ groups, which are contributed to the surface morphology. The SPEEK membranes showed that the hydrophilic nature of top surfaces is higher than that of the bottom surface, which is indicated by the angle of the water contact and stronger migration of cSMMs. The nodule structure on the membrane are shown in Figure 11. The water uptake versus methanol permeability and proton conductivity of blended membranes is shown in the Figure 12. 

**Figure 13:**
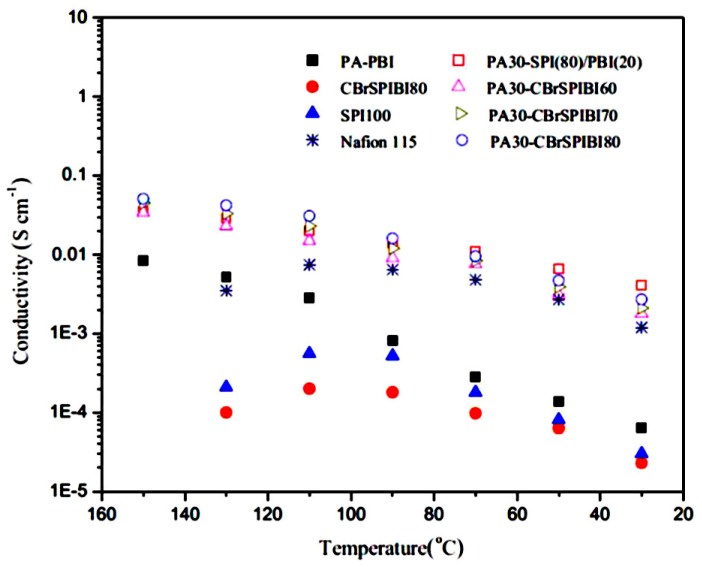
Temperature dependence of the conductivity of PA-CBrSPIBI, PA-SPI/PBI, CBrSPIBI80, SPI100, Nafion 115andPA-PBIat30%RH. Reproduced with permission from [100]. Copyright Elsevier, 2016.

The PEG200-HBS membrane of the smallest nodule size indicated the lowest water uptake and methanol permeability, whereas the largest nodule size indicated the highest conductivity and water uptake. The author concluded that the enhancement of proton conductivity was mainly due to the capability of the membrane which can absorb more water rather than the introduction of SO_3_ groups via cSMMs. Krishnan et al. [97] examined two polymers: a sulfonated polybenzimidazole homopolymer (MS-p-PBI 100) and a sulfonated poly(aryl ether benzimidazole) copolymers (MS-p-PBI 50, 60, 70, 80, 90), synthesized from available commercial monomers. The results indicated that sulfonated polybenzimidazole showed the properties of proton conductivity, thermal stability, and insolubility in boiling water. The combination of 1 wt % of MS-p-PBI 100 and 99 wt % of sulfonated poly ether sulfone 70 (PES 70) produced the blend membrane (BM) 1 which reduces membrane swelling; this can lead to the comparable proton conductivity and good dimensional stability. Hence, the authors claimed that BM 1 is for the fabrication of a membrane electrode assembly (MEA) in proton exchange membrane fuel cell (PEMFCs) and DMFC applications. Yang et al. [98] observed methanol permeability decreases and increases concentrations of methanol from 2.5 M to 20 M. This could be due to the incorporation of sulfonated cyclodextrin in SPEEK membranes. In this case, the proton conduction and proton conductivity increased upon introduction of sulfonated cyclodextrin. Further, the activation energy was calculated for proton conduction in the blend membranes. The results noted that the maximum value (4.20 kJ mol^−1^) was lower than Nafion^®^ 115 (9.15 kJ mol^−1^). Furthermore, the selectivity of the blend membranes, a compromise between proton conductivity and methanol permeability, was much higher than Nafion^®^ 115 at 15 wt % of the sulfonated cyclodextrin. The DMFCs polarizations were 2.5 M and 8.0 M methanol solutions with the variety of weight percentage (15, 20, and 25 wt %) of sulfonated cyclodextrin blend membranes were determined, respectively. The blend membrane with 20 wt % of sulfonated cyclodextrin showed the highest power density of 29.52 mW cm^−2^ at 120 mA cm^−2^ and 8.0 M methanol solution. The analysis suggests that the SPEEK membranes with the addition of sulfonated cyclodextrin in DMFCs can be used as potential usage applications. Jithunsa et al. [99] developed a sulfonated poly ether ether ketone (SPEEK) membranes containing poly(AA-co-4VIm) at a low humidity operation in the polymer electrolyte fuel cells. The sulfonated membranes showed an improvement in thermal stability compared with SPEEK membrane. The authors suggested that the ion-exchange capacity (IEC) and proton conductivity of the sulfonated membranes increase with the increase in the acrylic acid content in poly (AA-co-4VIm). However, the proton conductivity and the activation energy, decrease for SPEEK/M4. In the case of the higher content of imidazole, the membrane conductivity increased gradually up to a high temperature of 120 °C, especially for SPEEK/M2 and SPEEK/M3. The comprehensive performances of the composite membranes indicate that based on the swelling, thermal stability, mechanical properties, proton conductivity, ion exchange capacity, and hydrophilicity, the imidazole content of the sulfonated composite membranes ranged 30–60% for the poly(AA-co-4VIm). We understand that a polymer chain with both donor and acceptor functional groups play a major role in the improvement of proton conductivity as well as membrane properties. Yue et al. [100] fabricated alkaline imidazole sulfonated poly imide-benzimidazole (CBrSPIBIs) cross-linked with phosphoric acid (PA) using 4,4′-bibromomethenyl diphenyl ether as a cross-linker because of CBrSPIBIs cross-linked network can reduce the ionic conductivity (blocking of the hydrophilic channel). In Figure 13, we can see that the proton conductivity properties of all the systems are better than PA-CBrSPI and PA-SPI/PBI composite membranes when compared to CBrSPI and Nafion^®^ 115. Accordingly, the cross-linked network exhibited higher mechanical strength and better chemical stability, demonstrating the PA-CBrSPIBI membrane possibly being used as PEMs.

### 3.2. Sulfonated Inorganic Membranes

Chemical degradation of membranes is a major issue for fuel cell performance. In order to improve the long-term stability, the introduction of some inorganic compounds into the polymer composite membranes was considered as an alternative approach. The incorporation of inorganic fillers (SiO_2_, ZrO_2_, and TiO_2_) into polymer membrane can improve the thermal stability, mechanical properties, and proton conductivity at relatively high temperature (90–120 °C) [101,102,103,104]. A study by Changkhamchom et al. [105] examined the sulfonated blend membrane consisting of sulfonated poly ether ketone ether sulfone (SPEKES) and poly phenylene ether ether sulfone (PPEES) with different molar ratios (1:1, 2:1, 3:1, and 5:1) of SPEKES/PPEES. The author observed that the sulfonated blend membranes indicated the enhancement of higher methanol permeability and mechanical properties.

**Figure 14:**
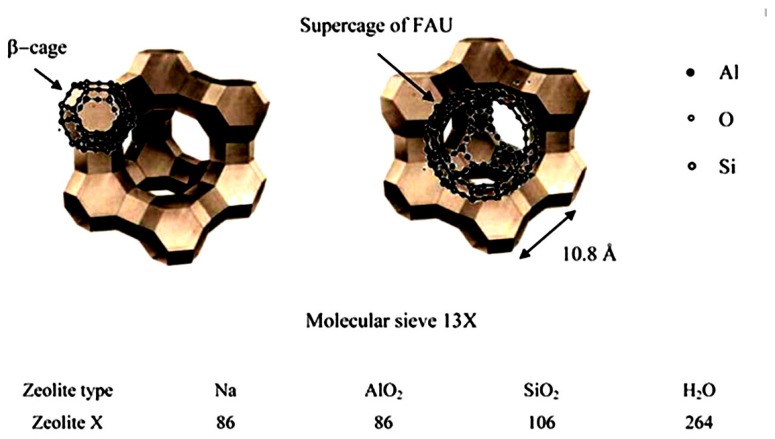
Structure of molecular sieve 13X. Reproduced with permission from [105]. Copyright Wiley, 2017.

**Figure 15:**
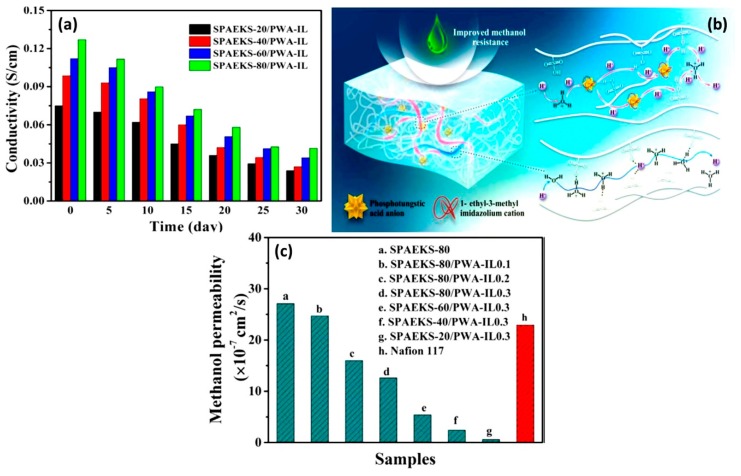
(**a**). The conductivity of SPAEKS-X/PWA-IL membranes at 80 °C during 30 days (**b**). Illustration of two proton transport mechanisms in SPAEKS-X/PWA-IL membranes. (**c**) Methanol permeability of composite membranes and Nafion^®^ 117 at room temperature. Reproduced with permission from [106]. Copyright Elsevier, 2017.

Figure 14 represents the structure of molecular sieve 13X/SPEKES. It was observed that the introduction of higher content of molecular sieve in 13X/SPEKES (15 % v/v) can favor the proton transfer along with the polymer chain, as well as the ionic interaction between hydrogen bonding jumping from sulfonic acid groups. Moreover, the selectivity of the membrane with (15% v/v) molecular sieve 13X/SPEKES/PPEES (5:1) composite membranes are 2.95 × 10^5^ Ss cm^−3^, which is a 123 higher than Nafion^®^ 117 and 3.6 times higher than the pristine SPEKES are presented in Table 1. Therefore, the compositing of SPEKES and PPEES can improve the permeability of methanol and the enhancement of the proton conductivity by incorporation of molecular sieve 13X. Li et al. [106] prepared sulfonated poly arylene ether ketone sulfone (SPAEKS) and 1-ethyl-3-methyl imidazolium phosphotungstate (PWA-IL). Their studies indicated that the addition of PWA-IL into the SPAEKS-X matrix enhanced proton conductivity and water uptake. The authors attributed the enhancement to protons that may have been transferred by vehicular mechanism and Grothus mechanism, which was confirmed by two different transport manners (Figure 15a,b). Conversely, SPAEKS-80/PWA-IL shows better methanol resistance than Nafion^®^ 117 (Figure 15c). Presumably, the organic–inorganic PWA-Il composite membrane could block the methanol penetration channel, consequently improving the methanol resistance of the composite membranes. The SPAEKS-X/PWA-IL composite membranes showed superior thermal stability when compared to the SPAEKS-X membranes. 

**Figure 16:**
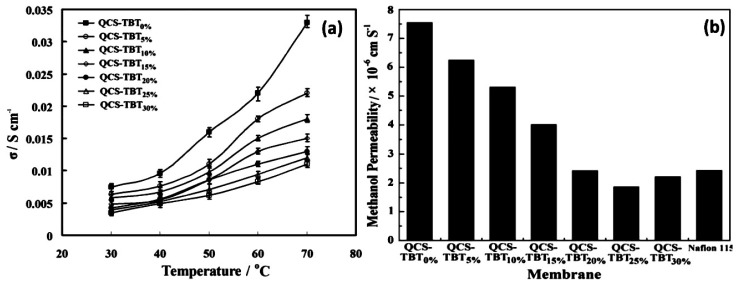
(**a**) Anionic conductivities of the QCS-TBTx% membranes (QCS-TBT0%, QCS-TBT5%, QCSTBT10%, QCS-TBT15%, QCS-TBT20%, QCS-TBT25%, and QCS-TBT30%) with different TBT content as a function of temperature; (**b**) methanol permeability of Nafion^®^ 115 and QCS-TBT_x%_ hybrid membranes (QCSTBT_0%,_ QCS-TBT_5%_, QCS-TBT_10%_, QCS-TBT_15%_, QCS-TBT_20%_, QCS-TBT_25%_, and QCS-TBT_30%_). Reproduced with permission from [107]. Copyright Elsevier, 2015.

Wang et al. [107] prepared an alkaline hybrid membrane through cross-linked quaternized chitosan with different contents of tetrabutyl titanate (TBT) for alkaline direct methanol fuel cells (ADMFCs). The anionic conductivity and methanol permeability of the hybrid membrane decreased with the increasing content of TBT, when compared to Nafion^®^ 115 (Figure 16a,b). These complications can be water retention ability at a high temperature of the inorganic dopant. These outcomes were revealed from water uptake properties. This is attributed to the hydrophobic/hydrophilic balance by changing crosslinked quaternized chitosan (QCS)/tetrabutyl titanate (TBT) ratio. The author has indicated that the inorganic network structure (TBT) enriches thermal stability and decreases conductivity, methanol permeability, and water uptake properties. Then more comprehensive properties than neat QCS membrane (QCS-TBT_0%_) were achieved. Shabanikia et al. [108] studied the perovskite-type SrCeO_3_ nanoparticles with polybenzimidazole (PBI) by solution casting technique. Proton conductivity increases with a filling of nanoparticles up to 8 wt % (PSC8). This may be possible by the hygroscopic nature of SrCeO_3_ nanoparticles in the membrane. At higher loads (>8 wt %) of SrCeO_3_ nanoparticles, the proton conductivity (PSC16) decreases due to self-aggregate of SrCeO_3_ nanoparticles. Moreover, the nanocomposites membrane showed a greater acid uptake, high thermal stability, and proton conductivity other than the PBI membrane. Bonis et al. [109] synthesized phenyl sulfonic functionalized nanometric titania (TiO_2_-PhSO_3_H) and introduced the filler of (Nafion^®^) developed a N_X (5, 10, and 20 wt %) into the TiO_2_-PhSO_3_H membrane-based composite membrane for DMFC application. The conductivity and methanol permeability of the N_10 wt % TiO_2_-PhSO_3_H membrane showed the highest value of 0.11 S cm^−1^ at 140 °C. Accordingly, methanol permeability (N_10 wt % TiO_2_-PhSO_3_H) 20% reduction of *J_lim_* and better than Nafion^®^. This is due to the chemical nature of the surface functionalization of TiO_2_-PhSO_3_H with enhanced DMFC’s performance. Pandey et al. [110] reinforced PVDF membrane with silica-immobilized phosphotungstic acid (Si-PWA/PVDF). Methanol permeability and proton conductivity of Si-PWA/PVDF membrane showed lesser value than Nafion^®^ 117 and 115. The water uptake Si-PWA/PVDF membrane displayed higher value than Nafion^®^ 117. The author exposed that the proton conductivity and methanol uptake of Si-PWA/PVDF membrane increases with increasing temperature. This is attributed to a smaller thickness of Si-PWA/PVDF membrane compared to Nafion^®^ 117. Pandey and Shukla et al. [111] prepared silica immobilized phosphotungstic acid (Si-PWA) for DMFCs. Proton conductivity is increased upon the addition of PWA (0.5 wt % to 1.5 wt %) due to the uniform dispersion of phosphotungstic acid (PWA) particles of silica network. The authors reported an improved single cell DMFCs performance when compared to Nafion^®^117 at 25 °C and 60% relative humidity. Zhong et al. [112] fabricated a multilayer membrane using silicon-containing sulfonated polystyrene/acrylate (SisPS/A) and chitosan (CS) using a deposition technique. The proton conductivity and water uptake decreased by the addition of C-SisPS/A-CS bilayers (5, 10, and 15). This could be that the multilayer membranes are blocked the charger carrier species besides the selectivity of C-SisPS/A-CS membrane (15). The membrane was 1.90 × 10^5^ Ss cm^−3^ compared to 1.9 times pure membranes and 8.9 times of Nafion^®^ 117. The obtained results indicated that the cross-linked multilayer membrane structures are promising materials for DMFC applications. Ahmad et al. [113] prepared polybenzimidazole (PBI), zirconium phosphate (1 wt % and 5 wt %) and Nafion^®^ using a casting method with dimethylacetamide. The dispersion of zirconium phosphate can decrease proton conductivity, methanol permeability, and selectivity up to 5 wt % when compared to 1 wt % (zirconium phosphate). It shows better performance than that of Nafion^®^ 117 (Table 1). The reason for proton donating SO_3_H is to reduce bonding and decrease the performance. The obtained results prove that the Nafion^®^-PBI-ZP hybrid membrane is better than Nafion^®^ 117. 

### 3.3. Sulfonated Organic–Inorganic Hybrid Membranes

**Figure 17:**
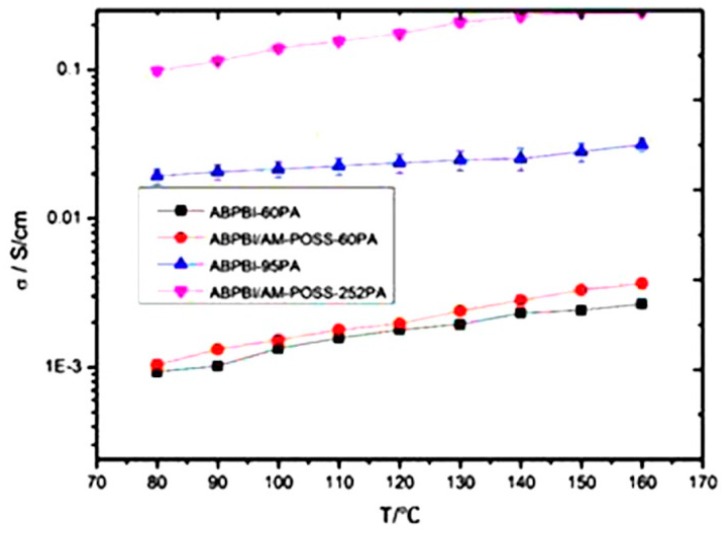
Proton conductivity of H_3_PO_4_-doped ABPBI and ABPBI/AM-POSS composite membranes at an anhydrous atmosphere. Reproduced with permission from [119]. Copyright Elsevier, 2016.

**Figure 18:**
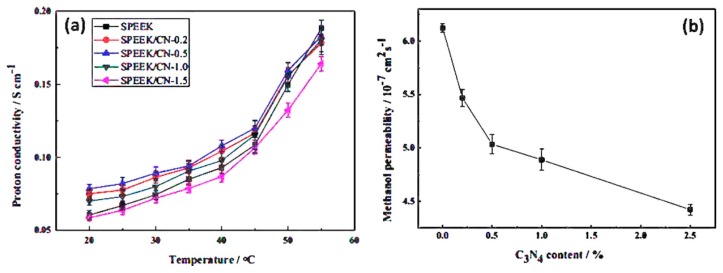
(**a**) Temperature-dependent proton conductivity of the membranes at 100% RH. (**b**) Methanol permeability. Reproduced with permission from [122]. Copyright Elsevier, 2016.

It is crucial that the organic filler should disperse uniformly within the polymer network and minimize the self–aggregation of inorganic additives in the polymer/inorganic membranes. Moreover, inorganic fillers should not leach out from the membrane matrix during long-time applications [114,115]. One of the best methods to overcome these problems is to prepare an organic–inorganic hybrid membrane via a sol-gel method. The sol-gel method can easily control the particle size distribution of the inorganic phase within the polymer network. Then, the network between organic and inorganic hybrid components can form within the matrix. It can be expected that the cross-linked organic and inorganic hybrid membrane will have a high proton conductivity, electrochemical properties, high selectivity and thermal stability [116]. Kim et al. [117] developed a sulfonated fluorinated block copolymer (SFBC-50) synthesized by casting method. The functionalized silica (FSiO_2_) composite 12 wt % membrane showed the best performance of proton conductivity and water uptake compared with neat SFBC-50 and Nafion^®^ 117 (Table 1). Furthermore, the reinforcement of FSiO_2_ particles, improves the thermal and mechanical properties. The authors claimed that out of the combination of higher bound water content and high per cluster volume of the SO_3_H groups, the FSiO_2_-X composite membrane exhibited better performance of proton conductivity cell under 40% relative humidity (RH) compared to the neat SFBC and Nafion^®^ 117 membranes. Han et al. [118] synthesized sulfonated poly arylene ether ketone sulfone (SPAEKS) polymer containing carboxyl groups (C-SPAEK) and 3-aminopropyl-triethoxysilane and tetraethoxysilane by the solgel method. The silica content of C-SPAEKS/KSiO_2_-8 (6.69 × 10^−7^ cm^2^ S^−1^) and C-SPAEKS/SiO_2_-8 (6.71 × 10^−7^ cm^2^ S^−1^) at 60 °C temperature owing to SiO_2_ particles act as a barrier to hinder the penetration of methanol. The cross-linked structure formed in the C-SPAEKS/KSiO_2_ membrane can block the penetration of methanol. Further, the proton conductivity of C-SPAEKS/KSiO_2_-8 (0.110 S cm^−1^) was greater than that of C-SPAEKS/SiO_2_-8 (0.082 S cm^−1^) when compared with the Nafion^®^ 117 (0.028 S cm^−1^) at the temperature of 120 °C observed and the details of conductivity values are given in Table 1. The author noted that the cross-linked hybrid membranes exhibited better performance in thermal stability and mechanical properties. Liu et al. [119] synthesized a hybrid composite poly 2,5-benzimidazole (ABPBI/Octa ammonium polyhedral oligosilsesquioxane (AM-POSS) using ABPBI with AM-POSS. The hybrid composites (ABPBI/AM-POSS) developed a doping method through solvent casting in phosphoric acid (PA). The improvements of proton conductivity owing to increasing temperatures as well as increasing of PA content are shown in Figure 17. However, the water uptakes of ABPBI/AM-POSS hybrid composite membranes were higher than the ABPBI and this may be due to the POSS dominant particles of water uptake. Further, the ABPBI/AM-POSS hybrid composite membranes with a higher level of PA doping indicated that these composites are very noble for applying in proton exchange membrane fuel cells (PEMFCs). Peng et al. [120] developed nano-hybrids by using graphene oxide (GO) through atom transfer radical addition (ATRA) reaction between C–F groups of Nafion^®^. The obtained results were that nanocomposite membranes (NM)/GO-0.05 and 0.10 (Table 1) showed higher proton conductivity, which is better than Nafion^®^ 212. This progress induced by the fabrication with the thermal extrusion process, and the microcrystalline domains of fluorocarbon present in the backbones of Nafion^®^ were poorly established in the cast of the Nafion^®^ membrane. In this study, the authors concluded that in order to obtain optimum conditions of the NM/GO nanocomposite membranes, further work needs to be carried out to enhance their properties. Feng et al. [121] investigated the imidazolium-based organic–inorganic hybrid alkaline anion exchange membrane (AEM) prepared via cross-linking of styrene, acrylonitrile, 1-vinyl-propyl-triethoxy silane imidazolium chloride (VPSIm)(Cl) and 1-vinyl-3-butylimdazolium- bromide (VBIm)(Br) for fuel cell applications. The hybrid membrane conductivity, temperature increases upon addition of AEMS (0, 5, 10, 15, 20 and 25 wt %). For example, the introduction of (VPSIm)(Cl) increases conductivity of the hybrid membrane-0 (3.58 × 10^−2^ S cm^−1^ at 30 °C) and membrane-5 (4.10 × 10^−2^ S cm^−1^ at 30 °C) (Table 1). Besides, decreased conductivity was observed with the addition of (VPSIm)(Cl) and this may be due to the inorganic network (Si–O–Si) in the membranes. Moreover, the water uptake of the hybrid membrane decreases with increasing content because of the inorganic network (Si–O–Si) in the hybrid membranes. The AEMS hybrid membranes (5, 10, 15, 20, and 25 wt %) showed good thermal stability, mechanical properties, and high conductivity. Gang et al. [122] studied the two-dimensional graphitic carbon nitride (g-C_3_N_4_) nanomaterial fabrication with SPEEK. The SPEEK/CN-0.2 nanocomposites membrane showed the highest proton conductivity (Figure 18a) among another membrane. The results indicated that the lower level of g-C_3_N_4_ contents increased the proton conductivity, which is mainly ascended from the prominent proton mobility because of 2D structural g-C_3_N_4_ nanosheets. However, the incorporation of g-C_3_N_4_ nanosheets in SPEEK reduced greatly the methanol permeability and the results are presented in Figure 18b. In Figure 18b, we can see the reduction in methanol permeability (27.8%) when compared to the SPEEK (6.12 × 10^−7^ cm^−2^ S^−1^) and SPEEK/CN-2.5 (4.42 × 10^−7^ cm^−2^ S^−1^). These changes are attributed to following issues (i) 2D g-C_3_N_4_ consisted of repeat triangular nanopores; and (ii) lower glass transition temperatures because of the mobility of the polymer (SPEEK) chains. The water uptake increases with increasing g-C_3_N_4_ nanosheets. The higher level contents of g-C_3_N_4_ nanosheets were unable to absorb excess water because the g-C_3_N_4_ nanosheets strongly suppressed the mobility of the SPEEK macromolecule. It concluded that these membranes exhibited superior thermal and mechanical properties and can be used for high-performance fuel cell application. 

**Figure 19:**
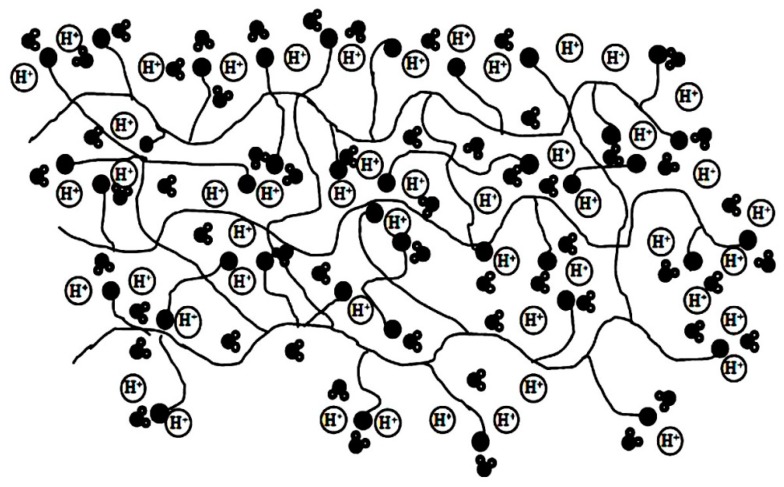
Illustration of the hybrid structure simulating the protons transfers. Reproduced with permission from [124]. Copyright Elsevier, 2015.

He et al. [123] improved proton exchange membranes (PEMs) prepared by sulfonated polyimide (SPI) and mesoporous organosilicate (MSiSQ) through sol-gel process. Proton conductivity and selectivity properties of all hybrid membrane increased with increasing temperature. Among all hybrid membranes, the best performance was achieved by SPI-40-MSiSQ compared with neat SPI and Nafion^®^ 117, respectively (Table 1). The thermal and mechanical properties were also enhanced due to the incorporation of MSiSQ in sulfonated polyimide (SPI). Consequently, the methanol permeability decreased with increasing the content of MSiSQ, because the introduction of polysiloxane network restricted the motion of polymer segments. The water uptake values of hybrid membranes are lower (15–18%) than the neat SPI, as a consequence of the microstructure and IEC of the hybrid membrane. The authors also indicated that the higher selectivity resulted from the improvement of proton conductivity and methanol permeability. Mosa et al. [124] also prepared an organic–inorganic hybrid membrane using 3-glycidoxypropyltrimethoxysilane (GPTMS) and 3-mercaptopropyl- trimethoxysilane (MPTMS) by the sol-gel method. The attained methanol permeability decreased upon loading the MPTMS content, indicating that MPTMS moieties could restrict the methanol permeation, and thus enable proton transport through sulfonic acid groups (Figure 19). For 60MPTMS-40GPTMS hybrid membrane, the conductivity increased up to 7.62 × 10^−2^ S cm^−1^ at 24 h. 

Hattori et al. [125] synthesized alkoxy derivative and F-substituted phenyl vinyl phosphonic acid via copolymerization by sol-gel technique. They prepared TMSMS/FC6 H4VPA with different ratios of 1/2, 1/4, and 1/6 (TMSMS/FC6H4VPA). Upon decreasing the Si/P ratio, they achieved a maximum conductivity of 6.4 × 10^−2^ S cm^−1^ (Si/p = 1/6) at 130 °C/100% RH, which indicated that the increasing content of phosphonic acid act as a proton carrier. In addition, the TMSMS/FC6H4VPA membrane exhibited good thermal stability and mechanical properties. The hybrid TMSMS/FC6H4VPA membranes supporting the growth of PEFCS can cover a board operating condition from low moisture to 100% RH. Prapainainar et al. [126] fabricated two types of coupling agents like γ-glycidoxylpropyl trimethoxy silane (GMPTS) and 3-mercaptopropyl triethoxysilane (MPTES) with surface modification using mordenite (MOR) to prepare Nafion^®^/MOR composites by solution casting process. The proton conductivity of the composite membrane MOR/MPTES displayed greater proton conductivity than MOR/GMPTS, and recast Nafion^®^. The proton conductivity of MOR/GMPTS membrane slightly decreases due to the increase in the amount of mordenite and the agglomeration processes in the mordenite of nanoparticles. Further, the increasing proton conductivity trend was absorbed in MOR/MPTES due to the increase concentrations of zeolite and sulfonic groups. Similar results were found in water uptake properties of MOR/MPTES. The higher water adsorption capacity is owing to the presence of a sulfonic group that acts as a hydrophilic group. Furthermore, GMPTS presented a higher methanol permeability compared to MPTES. This could be MPTES with good adhesion properties than GMPTS. The author found that the MOR/MPTES exposed a remarkable reduction of methanol permeability (5 wt %) and lower than MOR/GMPTS. Ahn et al. [127] prepared sulfonated poly phenylene oxide (SPPO) and hollow glass microspheres (HGMs) for DMFCs. The obtained results for SPPO-HGM 9 wt %/C-SPPO membrane showed lower methanol (2.67 × 10^−7^ cm^2^ s^−1^) permeability and higher proton conductivity (0.0278 S cm^−1^) compared with Nafion^®^ 117 (Table 1). Moreover, the incorporation of SPPO-HGM particles within the C-SSPO membranes enhanced both thermal and mechanical stability. Therefore, the SPPO-HGM 9 wt %/C-SPPO membranes can be used as PEMs for potential DMFCs applications. Zhang et al. [128] prepared dual cross-linked organic–inorganic hybrid membrane on bromomethylated poly ether ether ketone (BrPEEK), 3-aminopropyl triethoxysilane (APTES) with doping different weight percentage of phosphoric acid (PA). The incorporation of APTES (5, 10, 15, 20 and 30 wt %) within BrPEEK by doping PA, the proton conductivity increased by 10 wt% and the decreased at 30 wt % for all performances. The PA-QPEEK-10%APTES membrane showed the highest proton conductivity (Table 1) due to that the connectivity of PA cluster on the membrane surface influenced mainly the proton conductivity. PA-QPEEK-_X_%APTES membranes also exhibited an improved mechanical strength and oxidative stability. The author suggested that the dual cross-linked organic–inorganic hybrid system improved the performance of high temperature proton exchange membranes (HTPEM). Zhong et al. [129] analyzed the fabrication of poly vinyl alcohol (PVA) and poly methacrylic acid-2-acrylamido- 2-methyl-1-propanesulfimponic acid-vinyltriethoxysilicone (PMAV) in the applications of the direct methanol fuel cell. The authors observed that the proton conductivity and water uptake values were decreasing with the increase of PMAV (10, 20, 30 and 40) content with PVA when compared with Nafion^®^ 117. Nonetheless, the selectivity values of PVA/PMAV membrane showed an increasing and followed by a decrease of selectivity with further increasing PMAV content. Further, the authors claimed that the PVA/PMAV membrane has a low cost, admirable methanol barrier, high selectivity; good thermal stability may be a suitable electrolyte for DMFCs. Pan et al. [130] developed an organic–inorganic hybrid membrane prepared by sulfonated polyimides using benzimidazole (SPIBIs) with glycidyl ether of polyhedral oligomeric silsesquioxanes (G-POSS). Further, the authors observed that proton conductivity increases with increasing temperature. The enhancement of proton conductivity may be due to an excess amount of water engaged in the organic, inorganic hybrid membrane (SPIBIs/G-POSS). In this case of lower water uptake was examined in the organic, inorganic hybrid membrane this should restrict of G-POSS moiety cross-linked with SPIBIs. Furthermore, the author suggests that the optimization of a cross-linked membrane range from 10^−3^ to 10^−2^ S cm^−1^ and the optimization depends on the sulfonated polyimides containing benzimidazole group (SPIBI) degree. Wu et al. [131] reported the fabrication of amino acids through TiO_2_ functionalization using four types of amino acids (L-cysteine, O-phospho-L-serine, aspartic acid and histidine) with sulfonated poly ether ether ketone (SPEEK). From the data, the water uptake and methanol permeability slightly decreased upon addition of filler content (5, 10, 15, and 20 wt %). Because of the reduction of the ionic channel size, the membrane with 15 wt % additive exhibited the best performance owing to the more hydrophilic character of hydroxyl groups. In this case, proton conductivity increased following the order SPEEK/TiO_2_–Scys > SPEEK/TiO_2_–Pser > SPEEK/TiO_2_–Asp > SPEEK/TiO_2_–H is, due to the different proton acceptor-donor capability of amino acids. The authors concluded that the high-performance of hybrid PEMs with different amino acid functionalized TiO_2_ fillers tunes the characters of channels/pathways of proton conduction and methanol diffusion. Chen et al. [132] synthesized poly methyl methacrylate (PMMA)-SiO_2_-P_2_O_5_ and PMMA-SiO_2_-P_2_O_5_-ZrO_2_ hybrid membranes developed by the sol-gel method. For all hybrid membranes, the proton conductivity increased with increasing temperature from 30 °C to 90 °C. For example, the hybrid membranes such as 60PMMA-30SiO_2_-10P_2_O_5_ and 60PMMA-30SiO_2_-P_2_O_5_- ZrO_2_ showed lower proton conductivity by the loading of ZrO_2_ concentration. These changes can be the processes of surface and bulk transport of the hybrid membrane. In the case of water uptake, increases with decreasing content of SiO_2_, this could be higher hydrophilicity of phosphate other than silicate. The authors claimed that this hybrid membrane could be applicable for low-temperature fuel cells. Ren et al. [133] fabricated SPAES/SiO_2_ hybrid membranes by then reaction 3-isocyanatopropyl) triethoxysilane (ICPTES) with tetraethoxysilane (TEOS). A series of novel organic–inorganic hybrid proton exchange membranes were prepared from sulfonated poly arylene ether sulfone Am-SPAES/SiO_2_ hybrid membranes showed that the proton conductivities, methanol permeability and water uptake increase with increasing the content of SiO_2_ (3, 6 and 10 wt %). Nevertheless (Am-SPAES/10 wt % SiO_2_), decreases when compared with the other systems and Nafion^®^ 117 due to the dilution effect of the excess amount of SiO_2_. Presumably, the improvement of thermal and mechanical stability of Am-SPAES/SiO_2_ hybrid membranes is attributed by the introduction of SiO_2_ particles. 

Kim et al. [134] studied a proton-conducting phosphotungstic acid (PWA)/sulfonated fluorinated block copolymer composite membrane with reduced hydrogen permeability in polymer electrolyte fuel cells. The proton conductivity values increase with increasing temperature IN PWA membrane. This might be that the mobility of dynamics ions favor in increasing temperature and also act as the backbone of polymer structure to improve the ion conductivity at high temperature. Furthermore, sulfonated poly ether sulfone (SPES) membrane displayed 52 mS cm^−1^ (90 °C) and maximum proton conductivity was observed SPES/PWA-30 (116 mS cm^−1^ at 90 °C) which is similarly related to Nafion^®^ 117 (130 mS cm^−1^). The water uptake of SPES/PWA (10–30%) composite membranes decreased up to 10% and 20% other than 30% owing to the strong hydrogen bonding interaction between PWA and SPES. Mohanraj et al. [135] enhanced the properties of mechanical strength, thermal stability, and proton conductivity of ternary hybrid (SPEEK/SPVDF-HFP/GO) which was based on the membrane electrolyte in the applications of fuel cells. The ternary hybrid membranes, SPVdF-HFP increased the cluster volume of SO_3_H groups with an increase of the GO number of directional hydrogen bonds (H-bonds). The peak proton conductivity was observed at 90 °C attained by the SPEEK was 68 mS cm^−1^, a ternary hybrid was at 122 mS cm^−1^, 1.7 times better conductivity. A similar study by Mohanraj et al. [136] studied the improvement of mechanical strength, oxidative stability, and proton conductivity of an aligned quadratic hybrid (SPEEK/FPAPB/Fe_3_O_4_-FGO) membrane in the applications of high temperature and low humidity fuel cells. The hybrid Fe_3_O_4_-FGO improves proton conductivity, water absorption, and ion exchange capacity of the membrane by increasing the number of SO_3_H to retaining the stable dimension. Further, the results indicated that the peak proton conductivity of SPFSGF-5 the membrane at RH of 11.13 mS cm^−1^, and the temperature is about 120 °C. These values are 1.44-fold better than the SP membrane; whereas the Nafion^®^ 112 membrane exhibited a peak of proton conductivity at 9.78 mS cm^−1^ which was 1.13-fold lower. The authors noticed that the SPEEK/PWA and SPEEK/SPVdF-HFP/GO membranes are favorable for potential application in PEMFCs. 

## 4. Future Prospective

Various different s-Poly composites based on organic, inorganic, and organic–inorganic hybrid membranes for polymer electrolyte membranes have been reported extensively for use in direct methanol fuel cells to overcome the barrier issues encountered by Nafion^®^ at high temperatures and low humidity conditions. However, at high degrees, s-Poly may reduce the performance of PEM in DMFCs. Hence, several functionalization and modifications have been identified for rectification of the drawbacks in polymer blends. The modifications of polymer blends are: (i) a sandwiching polymer membranes technique; (ii) the optimization of lowering precious metal requirements for the improvement of power density; (iii) electrospinning processes for the improvement of DMFC performance; (iv) long-term operation and durability; and (v) the utilization of novel polymer blends and fillers along with the combinations of inventive layering techniques which could improve the cell membrane development in the field of high temperature fuel cell applications. These future aspects can then improve the DMFCs’ performance. 

## 5. Conclusions

We have hereby reviewed the present and future potential application of s-Poly-based composite and organic–inorganic hybrid material in order to improve the properties of PEM for direct methanol fuel cell applications. In this review, we comprehensively analyzed the chemical stability, proton conductivity, methanol permeability, selectivity, thermal and mechanical strength of different type of s-Poly composites and organic–inorganic hybrid membranes compared to commercially available Nafion^®^ membranes. The use of suitable compatibilizer and its optimum amount can result in better high proton conductivity, low methanol permeability, high selectivity, and thermal stability and mechanical properties from a vast array of literature. Most of the literature in this review reported that the s-Poly composite membranes, such as SPEEK, SPVdF, SPES, SPAES, SEBS, sPAEK, s-PSF, and S-PEKES are promising for direct methanol fuel cell applications. Especially, these s-Poly composites and organic–inorganic hybrid membranes provide increasing proton conductivity, selectivity, thermal stability, and mechanical properties and a decreasing methanol permeability compared to conventional Nafion^®^ membranes. An analysis of the literature shows that methanol permeability, proton conductivity and selectivity properties are the most significant in DMFCs applications. However, these properties cannot be used to predict with accuracy in the potential usage of a novel polymer. This might be due to the different aspects of stoichiometry and large oxygen in the presence of air at the cathode which can cause significant loss in fuel cell energy. Furthermore, this extensive review shows that the s-Poly composite and organic–inorganic hybrid membranes are potentially suitable for fuel cells applications at temperatures above 80–100 °C. Based on the data of the review, it can be concluded that the s-Poly composite and organic–inorganic hybrid membranes will further promote fuel cell related technology in future scenarios.

## Figures and Tables

**Table 1:** Data on methanol permeability, proton conductivity, and selectivity of s-Poly composite/organic–inorganic hybrid membranes compared to Nafion^®^ membranes.

Author	Blend Membrane Composition	Methanol Permeability (cm^2^ s^−1^) 10^−6^	Proton Conductivity (S cm^−1^)	Membrane Selectivity (Ss cm^−3^)
Bi et al.	cSPAES70-5/SPI(5/5)	-	11.1 **	-
Zhao et al.	SPEEK/PBa-15%	0.126 (at 30 °C) & 5.53 (at 80 °C)	4.25 × 10^−3^ (at 30 °C) & 2.46 × 10^−2^ (at 80 °C)	3.12 × 10^4^ (at 30 °C) & 4.45 × 10^3^ (at 80 °C)
Inan et al.	SPEEK70/PVDF(Mw¼275.000)	0.313 **	-	-
Unveren et al.	SPEES72/PVDF180 (10 wt %)	2.4 **	144.0 *	6.0 × 10^7^ *
Dutta et al.	PAni/PVdF-*co*-HFP/Pani	0.0000981 **	-	2.38 × 10^−6^
	PAni/SPVdF-*co*-HFP/PAni	0.0172 **	-	2.45 × 10^5^
	SPAni/SPVdF-*co*-HFP/SPAni	0.0529 **	-	9.39 × 10^4^
Seden et al.	SPEEK70/Copolymer 1a	0.082 **	84.4	-
	SPEEK70/Copolymer 1b	0.0013 **	30.5	-
Prasad et al.	SPEEK BNCM D-2	0.135 **	1.31 & 2.14 × 10^−3^	9.63 × 10^4^
Kumar et al.	S-20	1.76	3.16 × 10^−2^ *	1.80 × 10^4^
Mondal et al.	M2 (70/30 S-PVdF-*co*-HFP/PBI coated)	0.492 *	1.51 × 10^−2^ **	3.0695.3 *
Bagheri et al.	MSSP20	0.211	32.71	15.51 × 10^4^
Devi et al.	SPVdF-*co*-HFP/SPES blends	3.26 *	7.2 **	2.193 *
Molla and Compan	SPEEK-35%PVA	4.70 **	1.1 × 10^−2^ (at 120 °C) **	-
Muthumeenal et al.	SPES/NPHCs (1)	0.0171 *	9.2 × 10^−3^ (at 30 °C) * & 12.1 × 10^−3^ (at 80 °C)	5.3 × 10^4^ *
Meenakshi et al.	CS-PVA-SPES (25 wt %)	-	-	2.41 × 10^−4^ *
Norddin et al.	SPEEK/cSMM	0.275 **	6.4 × 10^−3^ *	-
Changkhamchom et al.	(15% v/v) Molecular sieve13X/S-PEKES/PPEES (5:1)	0.0487	1.44 × 10^−2^	2.95 × 10^5^
Ahmad et al.	Nafion-PBI 1%-ZP 1%	0.233927 **	0.02022 **	86,437.06 *
Kim et al.	FSiO_2_-12	-	100 **	-
Han et al.	C-SPAEKS/K-SiO_2_-8	0.667 (at 60 °C)	0.110 (at 120 °C) *	-
Peng et al.	NM/GO-0.10	-	40.8 (at 20 °C) 82.3 (at 95 °C)	-
Feng et al.	Membrane-5	-	4.10 × 10^−2^ (at 30 °C) 9.05 × 10^−2^ (at 90 °C)	-
He et al.	SPI-40-MsiSQ	0.018 **	0.566 (at 80 °C) *	12.8 × 10^6^ *
Ahn et al.	SPPO-HGM (9 wt %)/C-SPPO	0.267	0.0278	110,317.46 *
Zhang et al.	PA-QPEEK-10%APTES	-	61.7 (at 200 °C)	-

* Greater with respect to Nafion^®^; ** lesser with respect to Nafion^®^.

## Abbreviations

AEM	Alkaline anion exchange membrane
APTES	Aminopropyl Triethoxysilane
ATRA	Atom transfer radical addition
ADMFCs	Alkaline direct methanol fuel cells
Ba	Benzoxazine
BisPFPPO-OH	Bis ((p-hydroxy- tetrafluoro) phenyl) phenyl phosphine oxide
Bisphenol-AF	Bis (4-hydroxyphenyl) hexafluoropropane
BM	Blend membrane
BPPO	Bromomethylated poly phenylene oxide
BrPEEK	Bromomethylated poly ether ether ketone
cSMMs	Charged surface modified macromolecules
CS	Chitosan
CCSM 110	Chitosan Sulfate Composite Membranes
C30B	Cloisite 30B
cSPAES	Cross-linked sulfonated poly arylene ether sulfone
DCDPS	Dichlorodiphenyl sulfone
DS	Degree of sulfonation
DMFCs	Direct methanol fuel cells
FSiO_2_	Functionalization of silica
GA	Glutaraldehyde
GPTMS	Glycidoxy propyl trimethoxysilane
G-POSS	Glycidyl ether of polyhedral oligomeric silsesquioxanes
SGO	Sulfonated Graphene oxide
g-C3N4	Graphitic carbon nitride
HTPEM	High temperature proton exchange membranes
HGMs	Hollow glass microspheres
IEC	Ion-exchange capacity
ICPTES	Isocyanatopropyl triethoxysilane
MEAs	Membrane electrode assemblies
MPTES	Mercaptopropyl triethoxysilane
MPTMS	Mercaptopropyl- trimethoxysilane
MSiSQ	Mesoporous organosilicate
MOR	Mordenite
NM	Nanocomposites membranes
NMP	N-methyl-2-pyrrolidone
NPHCs	N-phthaloyl chitosan
OCV	Open circuit voltage
PEM	Polymer electrolyte membrane
PEMFCs	Proton-exchange membrane fuel cells
PA	Phosphoric acid
PBa	Polybenzoxazine
PWA-IL	Phosphotungstate
PWA	Phosphotungstic Acid
AMPS	Poly 2-Acrylamido-2-Methyl Propanesulfonic Acid
ABPBI	Poly 2,5-Benzimidazole
AM-POSS	Octa Ammonium Polyhedral Oligosilsesquioxane
PES 70	Poly Ether Sulfone 70
PFPPO-OH	Bis (P-Hydroxy- Tetrafluoro) Phenyl) Phenyl Phosphine Oxide
PPEES	Poly Phenylene Ether Ether Sulfone
PSSA	Poly Styrene Sulfonic Acid
PVA	Poly Vinyl Alcohol
PVDF	Poly Vinylidene Fluoride
PVDF-HFP	Poly Vinylidene Fluoride-Co-Hexafluoro Propylene
PBI	Polybenzimidazole
PES	Polyethersulfone
PEFC	Polymer Electrolyte Fuel Cell
PSSA	Polystyrene Sulfonic Acid
PU	Polyurethane
PCS	Polyvinyl Alcohol/Chitosan
PVB	Polyvinyl Butyral
PEMFCs	Proton Exchange Membrane Fuel Cells
PEMs	Proton Exchange Membranes
PMAV	Poly Methacrylic Acid-2-Acrylamido- 2-Methyl-1-Propanesulfimponic Acid-Vinyltriethoxysilicone
QCS	Quaternized Chitosan
RH	Relative Humidity
Semi-IPN	Semi-Interpenetrating Network
Si-PWA	Silica Immobilized Phosphotungstic Acid
SisPS/A	Silicon-Containing Sulfonated Polystyrene/Acrylate
SFBC	Sulfonated Fluorinated Block Copolymer
SGO	Sulfonated Graphene oxide
SPVDF-co-HFP	Sulfonated Poly Vinylidene Fluoride-Co-Hexafluoro Propylene
SPEES	Sulfonated Poly1,4-Phenylene Ether-Ether-Sulfone
SPAES	Sulfonated Polyarylene Ether Ketones
SPAEKS	Sulfonated Poly Arylene Ether Ketone Sulfone
SPEEK	Sulfonated Poly Ether Ether Ketone
SPEKES	Sulfonated Poly Ether Ketone Ether Sulfone
SPES	Sulfonated Poly Ether Sulfone
CBrSPIBIs	Sulfonated Poly Imide- Benzimidazole
SPPO	Sulfonated Poly Phenylene Oxide
sPAEK	Sulfonated Poly Arylene Ether Ketone
SPAni	Sulfonated Polyaniline
SPI	Sulfonated Polyimide
SPIBI	Sulfonated Polyimides Containing Benzimidazole
s-Poly	Sulfonated Polymer
SPSU	Sulfonated Polysulfone
SSA	Sulfosuccinic Acid
SPSF	Sulphonated Polysulphone
TBT	Tetrabutyl Titanate
TEOS	Tetraethoxysilane
VBIm-Br	Vinyl-3-Butylimdazolium- Bromide
VPSIm-Cl	Vinyl-Propyl-Triethoxy Silane Imidazolium Chloride
